# MEOX1 Coordinates Autocrine‐Paracrine Programs via SPHK1/S1P to Promote Lymph Node Metastasis in Ovarian Cancer

**DOI:** 10.1002/advs.202523574

**Published:** 2026-05-10

**Authors:** Jiajia Li, Xiuling Zhi, Qianhan Lin, Yating Sun, Yihua Sun, Zulimire Abudousalamu, Mengyang Xue, Chang Zheng, Xiaotian Li, Liangqing Yao, Mo Chen

**Affiliations:** ^1^ Department of Gynecologic Oncology Obstetrics & Gynecology Hospital of Fudan University Shanghai Key Lab of Reproduction and Development Shanghai Key Lab of Female Reproductive Endocrine Related Diseases Shanghai China; ^2^ Department of Physiology and Pathophysiology, School of Basic Medical Sciences Fudan University Shanghai China; ^3^ Department of Obstetrics and Gynecology Beijing Anzhen Hospital Capital Medical University Beijing China; ^4^ Department of Pathology Obstetrics & Gynecology Hospital of Fudan University Shanghai China; ^5^ Department of Obstetrics Shenzhen Maternity and Child Healthcare Hospital Shenzhen Guangdong China

**Keywords:** cancer‐associated fibroblasts, lymph node metastasis, MEOX1, ovarian cancer, SPHK1/S1P signaling

## Abstract

Lymph node metastasis (LNM) is a pivotal determinant of poor prognosis in ovarian cancer (OC), yet how tumor‐intrinsic programs remodel the microenvironment to enable spread remains unclear.Here, we identify the transcription factor mesenchymal homeobox 1 (MEOX1) as an upstream coordinator, whose overexpression associates with LNM, increased lymphatic density, and poor survival based on integrative analyses of public datasets and our 113‐patient cohort. In an in vivo LNM model, MEOX1 overexpression enhances tumor burden, lymphatic vessel density, and LNM, whereas tumor‐conditioned medium does not directly activate lymphatic endothelial cells (LECs), implicating stromal intermediates. Spatial transcriptomic and immunostaining analyses confirmed cancer‐associated fibroblast (CAF)‐LEC proximity and vascular endothelial growth factor‐C (VEGF‐C) localization within CAFs, supporting a CAF‐dependent lymphangiogenic route. Mechanistically, MEOX1 binds the sphingosine kinase 1 (SPHK1) promoter to activate sphingosine‐1‐phosphate (S1P) synthesis, driving a dual autocrine‐paracrine program: sphingosine‐1‐phosphate receptor 3 (S1PR3)‐dependent signaling promotes tumor proliferation/migration, while S1P/S1PR1 reprograms fibroblasts into VEGF‐C‐secreting, alpha‐smooth muscle actin (α‐SMA)‐positive CAFs that stimulate lymphangiogenesis and LNM; SPHK1 inhibition blunts these phenotypes, whereas S1P supplementation restores them. These findings provide novel insights into lymphatic metastasis and demonstrate that metastatic competence depends not only on intrinsic tumor aggressiveness but also on the acquired ability to construct a pro‐dissemination niche.

## Graphical abstract

In ovarian cancer, MEOX1 activates the SPHK1/S1P pathway to promote both tumor progression and tumor–stroma crosstalk. MEOX1‐dependent signaling drives CAF activation, enhances VEGF‐C expression, and stimulates lymphangiogenesis, ultimately facilitating lymph node metastasis. These findings identify MEOX1 as a key regulator of metastatic niche formation and a potential therapeutic target.

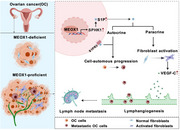

## 1 Introduction

Lymph node metastasis (LNM) is a pivotal event in the progression of solid malignancies, serving as a critical gateway for further dissemination and a powerful indicator of poor prognosis in various cancers, including ovarian cancer (OC) [1, 2]. This metastatic cascade is critically initiated by lymphangiogenesis, the formation of new lymphatic vessels from pre‐existing ones [3, 4]. Structurally, these neo‐lymphatic vessels are more permissive to tumor cell intrusion than blood vessels due to their larger lumens and discontinuous basement membrane [4]. Clinically, the density of these nascent vessels in both intra‐ and peri‐tumoral regions strongly correlates with metastatic frequency and reduced patient survival across multiple cancer types [5, 6, 7, 8]. Consequently, inhibition of tumor‐induced lymphangiogenesis has emerged as a promising therapeutic strategy. However, the mechanisms underlying lymphangiogenesis remain poorly understood, highlighting the urgent need for research to inform novel clinical interventions.

The establishment of LNM depends not only on the intrinsic invasiveness of tumor cells but also on the dynamic remodeling of the tumor microenvironment (TME) [9]. Within the TME, cancer‐associated fibroblasts (CAFs) have emerged as a central stromal effector, driving pro‐metastatic reprogramming [10, 11]. Supporting this, CAF abundance positively correlates with elevated lymphatic vessel density, LNM incidence, and poorer patient prognosis across diverse solid tumors, such as OC [8], esophageal cancer [12], bladder cancer [13], and breast cancer [14]. Co‐injection of CAFs with tumor cells in various xenograft models significantly enhances tumor growth, lymphangiogenesis, LNM, and distant metastasis in vivo [12, 15, 16, 17, 18]. Although evidence indicates that CAFs and lymphatic endothelial cells (LECs) form a dynamic stromal network that can strongly influence metastatic behavior, the tumor‐intrinsic signals that orchestrate CAF activation, VEGF‐C production, and lymphangiogenic niche formation remain largely elusive. Specifically, it is unknown whether a single upstream regulator can couple tumor‐cell transcriptional activity with stromal programs required for lymphatic spread.

Mesenchymal homeobox 1 (MEOX1), a member of the homeobox gene family, was initially mapped to the 17q21 region near the breast cancer susceptibility gene 1 (BRCA1) [19]. Our previous work identified MEOX1 as a transcription factor associated with LNM in OC [20], yet the underlying mechanism remained unclear. It is well established for its critical functions in embryonic development [21], Klippel–Feil syndrome [22], fibrosis [23, 24, 25], vascular remodeling [26], and tumor progression [27, 28, 29, 30, 31, 32]. This unique functional profile in fibrosis and vascular remodeling suggested that MEOX1 may serve as a key coordinator behind the stromal crosstalk and lymphangiogenesis central to metastasis. These led us to investigate whether MEOX1 serves as the transcriptional hub that provides the unifying mechanism for coupling the seemingly distinct processes of tumor cell invasion and stromal remodeling.

This study aimed to identify the tumor‐intrinsic regulator that enables ovarian cancer cells to remodel the stromal‐lymphatic microenvironment to support metastatic dissemination. Here, we show that MEOX1 integrates tumor‐intrinsic transcriptional activation with SPHK1/S1P lipid signaling and stromal reprogramming, forming a multi‐compartment axis that drives the construction of a lymphangiogenic niche. Our findings support that metastatic competence arises not only from reinforced tumor‐intrinsic aggressiveness but also from the acquired ability of cancer cells to actively construct a pro‐dissemination microenvironment.

## 2 Results

### 2.1 Clinical Profiling Identifies MEOX1 as a Lymphangiogenic and Prognostic Determinant in Ovarian Cancer

To identify LNM‐related genes in OC, we analyzed data from The Cancer Genome Atlas (TCGA) and four Gene Expression Omnibus (GEO) datasets (GSE69428, GSE18520, GSE54388, and GSE27651). By combining upregulated LNM‐related differentially expressed genes (DEGs) from TCGA and upregulated cancer‐related DEGs from GEO, we identified 13 candidate genes (Figure S1A,B). Prognosis evaluation revealed that elevated expression of *MEOX1*, secreted phosphoprotein 1 (*SPP1*), and protocadherin beta 2 (*PCDHB2*), was associated with shortened overall survival (OS) and progression‐free survival (PFS) in OC patients (Figure S1C). Among these, *MEOX1* exhibited the highest fold change (FC) between the groups with negative (LNM (−)) or positive (LNM (+)) lymph node metastasis status (Table S1).

**FIGURE 1:**
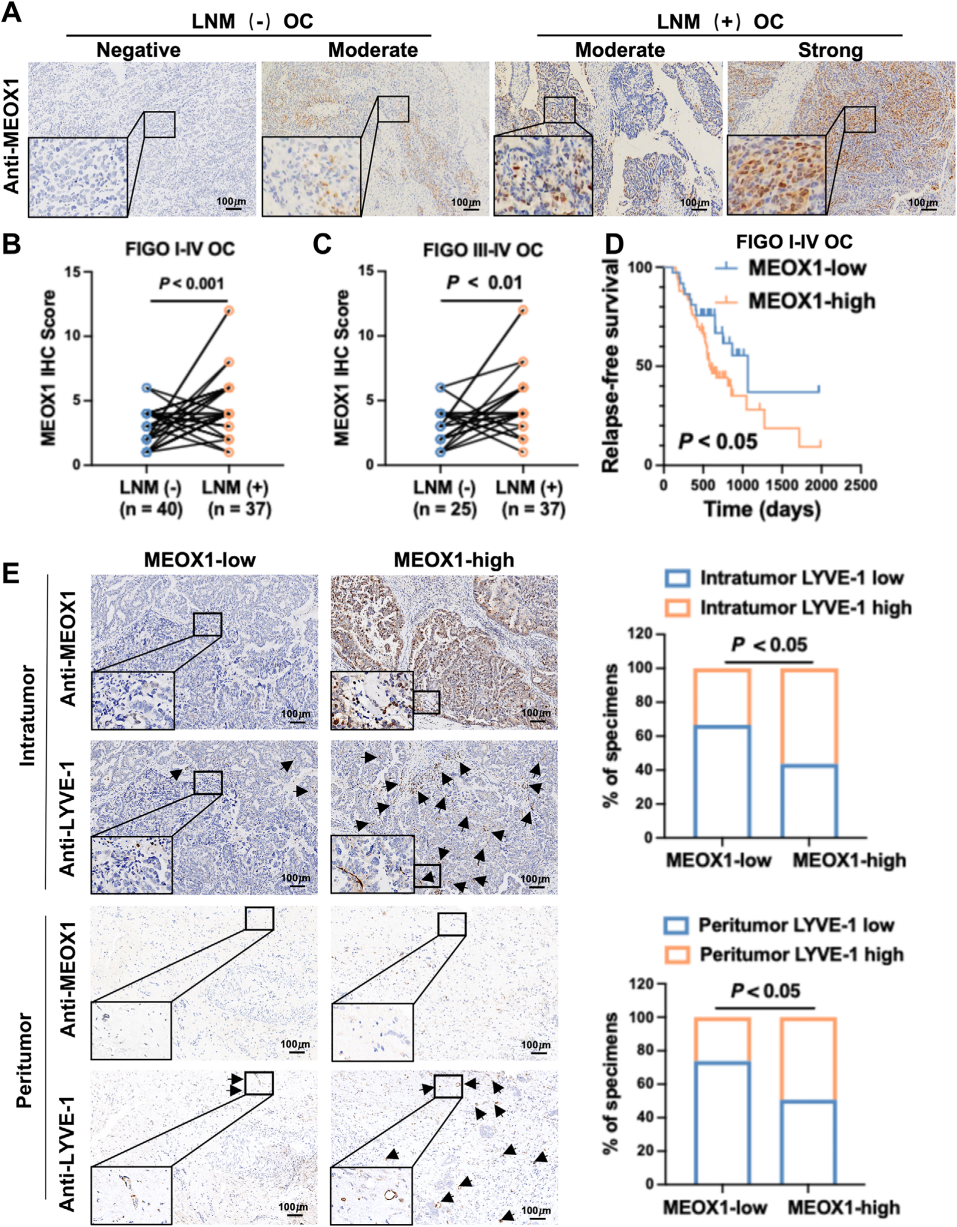
The expression of MEOX1 in ovarian cancer tissues was associated with lymph node metastasis and the density of neonatal lymphatic vessels. (A) Representative IHC staining images of MEOX1 in OC tissue samples with negative (LNM (−), *n* = 40) or positive (LNM (+), *n* = 37) lymph node metastasis status. (B) The statistical results of MEOX1 IHC scores in 40 LNM (−) and 37 LNM (+) OC specimens with FIGO I‐IV. (C) The statistical results of MEOX1 IHC scores in 25 LNM (‐) and 37 LNM (+) OC specimens with FIGO III‐IV. (D) The Kaplan–Meier survival curve between MEOX1 IHC scores and RFS in 104 OC patients with complete follow‐up information. The differences in survival between the groups were assessed using the log‐rank test. (E) Representative IHC staining images (*left*) and quantitative data of IHC scores (*right*) of LYVE‐1 and MEOX1 in intra‐tumoral and peri‐tumoral tissues of 113 clinical OC tissue specimens. Arrows indicate neonatal lymphatic vessels (LYVE‐1+). (LYVE‐1‐high: >8 tubular structures per field; LYVE‐1‐low: ≤8 tubular structures per field (100×), averaged over 3–5 hotspot fields per case; blinded double‐reading). Statistical analysis was performed by the Chi‐squared test.

To validate these bioinformatic findings, we assessed MEOX1 protein expression in a clinical cohort of 113 OC patients by immunohistochemistry (IHC). Of the 113 patients, 77 underwent lymph node dissection or biopsy, of whom 40 were LNM (−) (15 stage I‐II, 25 stage III‐IV) and 37 were LNM (+), based on nodal status. As shown in Figure 1A, MEOX1 exhibited predominant nuclear localization and markedly stronger staining in LNM (+) specimens compared with LNM (−) cases. Receiver operating characteristic curve (ROC) analysis demonstrated that the diagnostic AUC of MEOX1 was 0.73 (95% confidence interval (CI): 0.64–0.82), supporting its potential as a biomarker for LNM involvement in OC (Figure S1D).

All patients were subsequently stratified into MEOX1‐high (*n* = 71, IHC score ≥ 4) and MEOX1‐low (*n* = 42, IHC score < 4) groups. High MEOX1 expression correlated with advanced FIGO stage and the presence of LNM, but not with patient age, histological subtype, tumor size, CA125 levels, or the presence of ascites (Figure 1B, Table 1). To control for potential confounding by FIGO stage, we restricted subsequent analyses to patients with FIGO III/IV. The LNM (+) and LNM (−) groups showed balanced baseline clinicopathological characteristics, including age, FIGO stage, pathological tissue type, tumor size, CA125 level, and ascites status (Table S2). Within this cohort, MEOX1 IHC scores were significantly elevated in LNM (+) patients compared to LNM (−) controls (*p* < 0.01, Figure 1C). Moreover, high MEOX1 expression was associated with shorter relapse‐free survival (RFS) in OC patients (*p *< 0.05; Figure 1D).

**TABLE 1:** Correlation analysis between MEOX1 expression level and clinical pathological characteristics of ovarian cancer patients.

Variable	MEOX1‐low (*n* = 42)	MEOX1‐high (*n* = 71)	*P* ^a^
	*n* (%)	*n* (%)	
Age (years)			0.796
<50	14 (33.3)	22 (31.0)	
≥50	28 (66.7)	49 (69.0)	
FIGO stage			<0.001
I‐II	15 (35.7)	7 (9.9)	
III‐IV	27 (64.3)	64 (90.1)	
Histological subtype			0.095
Serous Adenocarcinoma	30 (71.4)	60 (84.5)	
Others	12 (28.6)	11 (15.5)	
Tumor size (cm^3^)			0.414
<1000	35 (83.3)	63 (88.7)	
≥1000	7 (16.7)	8 (11.3)	
Lymph node metastasis			<0.001
Absent	26 (74.3)	14 (33.3)	
Present	9 (25.7)	28 (66.7)	
CA125 level (U/mL)			0.130
<600	28 (66.7)	37 (52.1)	
≥600	14 (33.3)	34 (47.9)	
Ascites			
Absent	11 (26.2)	18 (25.4)	0.922
Present	31 (73.8)	53 (74.6)	

^a^ Chi‐square test.

To further assess the histological basis of lymphatic remodeling, we quantified lymphatic vessel endothelial receptor‐1 (LYVE‐1) positive vessels in ovarian cancer tissues. LYVE‐1, a well‐established marker for LECs, was used to delineate newly formed lymphatic structures [9]. We observed significantly higher densities of LYVE‐1+ vessels in both intra‐ and peri‐tumoral regions of MEOX1‐high ovarian cancer compared to MEOX1‐low cases (*p* < 0.05, Figure 1E), consistent with established associations between lymphangiogenesis and metastatic progression [5, 6, 7, 8]. These findings establish MEOX1 expression as a marker of aggressive disease characterized by enhanced lymphangiogenesis and lymphatic spread in OC.

### 2.2 MEOX1 Enhances Lymphatic Dissemination In Vivo Independent of Direct Tumor‐Lymphatic Endothelial Interaction

**FIGURE 2:**
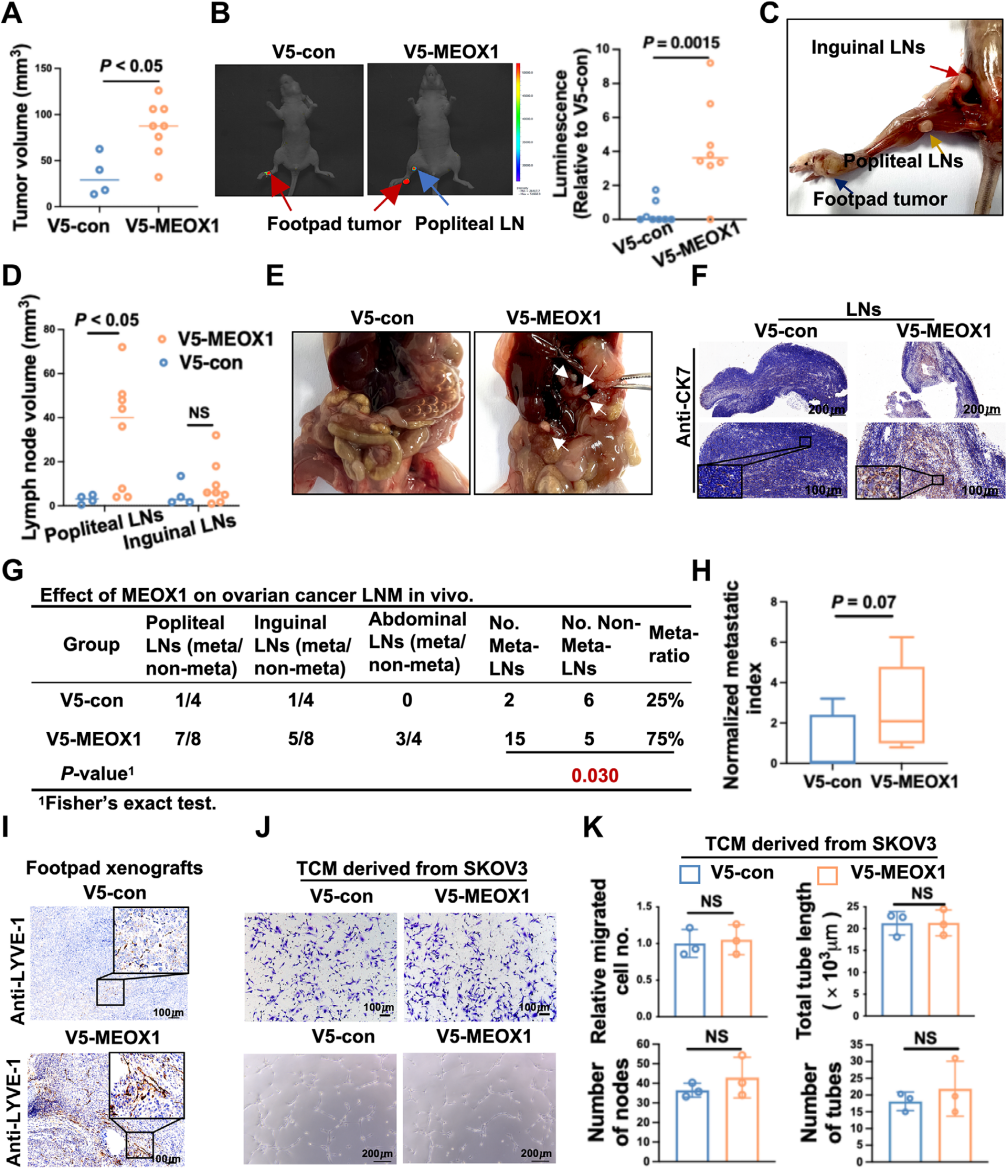
MEOX1 overexpression in ovarian cancer cells promoted LNM and lymphangiogenesis in vivo despite no direct effect on LEC migration and tube formation in vitro. (A) Tumor volumes of footpad xenografts at the endpoint of the experiment for V5‐con and V5‐MEOX1 groups. Data are presented as mean ± SD. (B) In vivo imaging of tumor growth and metastasis. Left panel: Representative in vivo fluorescence images of nude mice with inoculation of green fluorescent protein (GFP)‐labeled V5‐MEOX1 or control (V5‐con) SKOV3 cells into the footpads. Right: Quantification of GFP fluorescence intensity in the two groups. Data are presented as mean ± SD. (C) Representative photographs of footpad xenografts, popliteal LNs, and inguinal LNs. (D) Bar graph showing quantitative analysis of the volume of popliteal and inguinal LNs from the V5‐MEOX1 and V5‐con groups. Data are presented as mean ± SD. (E) Images of abdominal LNs in two groups of nude mice. White arrows point to enlarged abdominal LNs in the V5‐MEOX1 group. (F) Representative IHC staining images of CK7 in LNs of the two groups. (G) Statistical data of LNM status (Fisher's exact test). (H) Normalized metastatic index (number of metastatic lymph nodes/ primary tumor volume (mm^3^)) in V5‐con and V5‐MEOX1 groups. (I) Representative IHC staining images of LYVE‐1 in footpad xenografts of the two groups. (J) Representative images of Transwell migration assays and tube formation assays showing migrated HLECs and formed tubes of HLECs after 24 h of treatment with TCM from V5‐MEOX1 or control (V5‐con) SKOV3 cells. (K) Quantitative analysis of the number of migrated HLECs per field, and total tube length, number of tubes, and number of nodes of HLECs from the experiments shown in (J). Data are presented as mean ± SD from three independent experiments. NS, no statistical difference.

To investigate the functional contribution of MEOX1 to LNM in OC, we established a popliteal lymph node metastasis model using SKOV3 cell lines (which exhibited low endogenous MEOX1 expression) with stable MEOX1 overexpression (V5‐MEOX1) or empty vector control (V5‐con) (Figure S2). Overexpression of MEOX1 markedly increased footpad xenograft formation (Figure S3A,B), tumor growth rate (Figure S3C), and tumor volume (Figure 2A). In vivo fluorescence imaging revealed significantly increased tumor metastasis susceptibility in the V5‐MEOX1 group, as evidenced by a markedly higher mean fluorescence intensity compared with the control group (Figure 2B).

Following meticulous dissection, the lymph nodes (LNs) were photographed, and their dimensions were measured (Figure 2C). The volume of the popliteal LNs in the V5‐MEOX1 group was substantially larger than that in the control group. In contrast, the volume of inguinal LNs did not differ significantly between the groups (Figure 2D). Furthermore, four enlarged LNs were observed in the abdominal cavity of one nude mouse in the V5‐MEOX1 group (Figure 2E), whereas no enlarged abdominal LNs were observed in the control group. To determine whether these enlargements represented metastases, we performed IHC staining for cell keratin 7 (CK7), an epithelial cancer‐specific marker that is not expressed in normal LNs [33, 34, 35] (Figure 2F). Statistical analysis revealed that the overall LNM rate in the V5‐MEOX1 group was markedly higher than that in the control group (75% vs. 25%, Figure 2G). To exclude the possibility that MEOX1‐facilitated lymph node metastasis is an indirect consequence of enhanced primary tumor growth rather than a direct pro‐metastatic effect, we normalized metastatic burden to primary tumor volume by calculating a metastasis index (number of metastatic LNs / primary tumor volume in mm^3^). After normalization, the V5‐MEOX1 group still exhibited a higher metastatic index than the control group, with a clear trend apparent despite the difference not reaching statistical significance (*p* = 0.07, Figure 2H). These findings indicate that the increased LNM observed upon MEOX1 overexpression is likely attributable, at least in part, to a genuine pro‐metastatic effect, rather than being solely a passive consequence of increased primary tumor volume. Additionally, the density of neonatal lymphatic vessels in the footpad xenografts of the V5‐MEOX1 group was notably greater than that of the control group, with a *p* value approaching 0.05 (Figure 2I; Figure S3D). These results suggest that MEOX1 not only promotes tumorigenesis, growth, and LNM of OC cells in vivo but also favors lymphangiogenesis in tumor tissues.

To determine whether MEOX1‐driven lymphangiogenesis results from direct communication with lymphatic endothelial cells (LECs), we examined pro‐lymphangiogenic factors and performed functional co‐culture assays. Prolymphangiogenic factors, such as VEGF‐C, VEGF‐D, and platelet‐derived growth factor (PDGF), are involved in the proliferation, migration, budding, and tube formation of LECs [3, 9, 36]. We observed that modulation of MEOX1 expression in tumor cells did not alter *VEGFC* (Figure S4A), *VEGFD* (Figure S4B), or other prolymphangiogenic factors, including *PDGFA*, *PDGFB*, *PDGFC*, *PDGFD*, transforming growth factor β1 (*TGFB1*), *TGFB2*, *TGFB3*, fibroblast growth factor 1 (*FGF1*), *FGF2*, insulin‐like growth factor 1 (*IGF1*), and *IGF2* (Figure S4C). Notably, tumor cell‐derived culture medium (TCM) from V5‐MEOX1 SKOV3 cells failed to significantly enhance human LECs (HLECs) migration or tube formation in vitro (Figure 2J,K). Collectively, these findings demonstrate that OC cell‐intrinsic MEOX1 enhances tumor growth, lymphangiogenesis, and LNM in vivo, but not through direct tumor‐LEC interactions, implying that stromal intermediates may mediate this effect.

### 2.3 Spatial and Histological Analyses Reveal a Correlation of CAFs’ Infiltration With MEOX1 Expression and Lymphangiogenesis in OC

**FIGURE 3:**
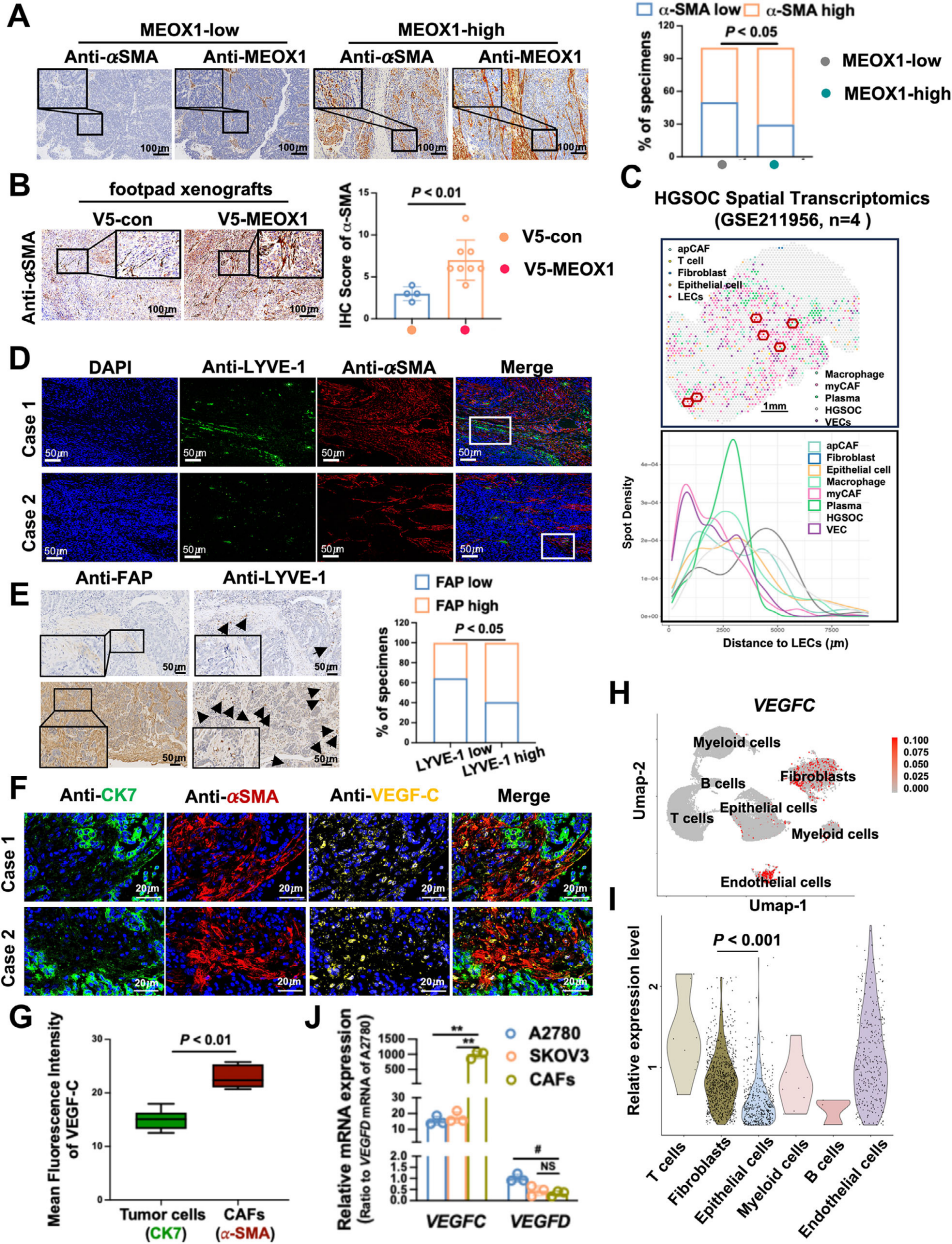
CAFs’ infiltration was correlated with MEOX1 expression and lymphangiogenesis in ovarian cancer. (A) Representative IHC staining images (*left*) and statistical data for IHC scores (*right*) of MEOX1 and α‐SMA in 113 OC tissues (α‐SMA‐high: IHC score > 6; α‐SMA‐low: IHC score ≤ 6) (Chi‐squared test). (B) Representative IHC staining images of α‐SMA (*left*) and statistical data of α‐SMA IHC scores (*right*) in footpad xenografts derived from the V5‐MEOX1 and the V5‐con SKOV3 cells in the nude mice popliteal LNM model for OC. (C) The distinct spatial distribution of key cell populations (upper) and the density–distance distribution of different cell types relative to LECs (lower) in the spatial transcriptomic analysis of four high‐grade serous ovarian cancer (HGSOC) samples (GSE211956). VECs, vascular endothelial cells. Scale bar: 1 mm. (D) Immunofluorescence analysis of human OC tissue sections stained for LYVE‐1 (a specific marker for LECs, green), α‐SMA (CAF marker, red), and DAPI (nuclei, blue). Case 1: a specimen with high MEOX1 expression. Case 2: a specimen with low MEOX1 expression. (E) Representative IHC staining images (*left*) and statistical data for IHC scores (*right*) of FAP (CAFs marker) and LYVE‐1 (HLECs marker) in 113 OC specimens. Specimens were stratified into FAP‐high (IHC score > 6) and FAP‐low (IHC score ≤ 6) groups (Chi‐squared test). (F) Representative multiplex immunofluorescence images of CAF‐enriched OC tissues. Tissue sections were stained with anti‐CK7 (green, tumor cell marker), anti‐α‐SMA (red, CAF marker), and anti‐VEGF‐C (yellow). Nuclei were counterstained with DAPI (blue). Scale bar, 20 µm. (G) Quantitative analysis of VEGF‐C expression in CK7‐positive tumor cells (green) versus α‐SMA‐positive CAFs (red) in OC tissues (*n* = 3). (H) UMAP plot of scRNA‐seq data from 17 OC patients (GSE151214, GSE154600), showing *VEGFC* expression across major cell types (T cells, fibroblasts, epithelial cells, myeloid cells, B cells, and endothelial cells). (I) Quantification of *VEGFC* expression across different cell types. (J) RT‐qPCR analysis of mRNA levels of *VEGFC* and *VEGFD* in A2780 and SKOV3 ovarian cancer cells and primary CAFs from human OC tissues. Data are presented as mean ± SD from three independent experiments. NS, no statistical difference; **p < 0.01, #*p *< 0.05.

Given that MEOX1‐driven lymphangiogenesis in vivo was not mediated by direct tumor cell‐LEC interaction, we next explored whether stromal components contribute to this process. In the TCGA OC cohort, MEOX1‐high cases exhibited enhanced CAF signatures, including elevated FAP, ACTA2, PDGFRB, and S100A4 expression (Figure S5A), and higher CAF infiltration scores across multiple algorithms (Figure S5B). Histologically, MEOX1‐high clinical OC specimens and footpad xenografts in the popliteal lymph node metastasis model displayed greater α‐smooth muscle actin (α‐SMA, a marker of CAFs) [37] positivity (Figure 3A,B).

In spatial transcriptomic analyses, we found that myofibroblastic CAFs (myCAFs), a subset characterized by high α‐SMA expression and robust extracellular matrix (ECM) remodeling capacity [38, 39], were enriched in close proximity to LECs (Figure 3C). Dual immunofluorescence staining for LYVE‐1 and α‐SMA also revealed close spatial proximity between HLECs and CAFs within the OC stroma, particularly in MEOX1‐high tumors (Figure 3D). Quantitative analysis confirmed elevated CAF infiltration in LYVE‐1‐high OC specimens (Figure 3E), which is consistent with previous reports [40]. Moreover, TCGA survival analysis revealed that OC patients with higher CAF infiltration had significantly shorter overall survival (OS) (Figure S6A).

To evaluate whether CAFs supply pro‐lymphangiogenic cues, we assessed VEGF‐C and VEGF‐D expression in OC, which were well‐established key mediators in lymphangiogenesis [36]. Multiplex immunofluorescence staining for anti‐CK7 (green, tumor cells), anti‐α‐SMA (red, CAFs), and anti‐VEGF‐C (yellow) was performed on CAF‐enriched OC tissues. As shown in Figure 3F, strong VEGF‐C staining was observed in α‐SMA‐positive CAFs, as evidenced by the yellow signal co‐localized with red staining. In contrast, CK7‐positive tumor cells exhibited relatively weak VEGF‐C expression. Quantitatively, the mean fluorescence intensity of VEGF‐C was significantly higher in CAFs than that in tumor cells (Figure 3G). This finding was further substantiated at the transcriptomic level through analysis of single‐cell RNA‐sequencing (scRNA‐seq) data from two independent cohorts (GSE151214, GSE154600; 17 ovarian cancer samples in total), which demonstrated a consistent and significant upregulation of VEGF‐C in fibroblasts compared to tumor cells (Figure 3H,I). Similarly, RT‐qPCR revealed markedly higher *VEGFC* mRNA levels in primary CAFs isolated from primary tumor tissues of OC patients compared with SKOV3 and A2780 OC cell lines (Figure 3J). In contrast to VEGF‐C, VEGF‐D expression showed no significant difference between tumor cells and CAFs (Figure 3J; Figure S6B). These findings establish CAFs as the primary source of VEGF‐C in the ovarian cancer microenvironment, positioning them as a key mediator of lymphangiogenesis.

To validate the role of CAFs in MEOX1‐driven lymphangiogenesis, we isolated primary CAFs from OC tissues and stratified them into MEOX1^high^ and MEOX1^low^ groups based on endogenous MEOX1 expression levels in tumors. Co‐culture with HLECs revealed that primary CAFs isolated from MEOX1^high^ OC samples significantly promoted HLEC tube formation compared with those from MEOX1^low^ samples, phenocopying the effect of NFs activated by MEOX1‐overexpressing SKOV3 conditioned media (Figure S6C). Together, these results indicate that MEOX1 expression in tumor cells confers a pro‐lymphangiogenic capacity to CAFs as a pivotal functional link between MEOX1‐overexpressing tumors and lymphatic metastasis.

### 2.4 MEOX1 Transcriptionally Activates the SPHK1/S1P Axis to Drive Ovarian Cancer Aggressiveness

**FIGURE 4:**
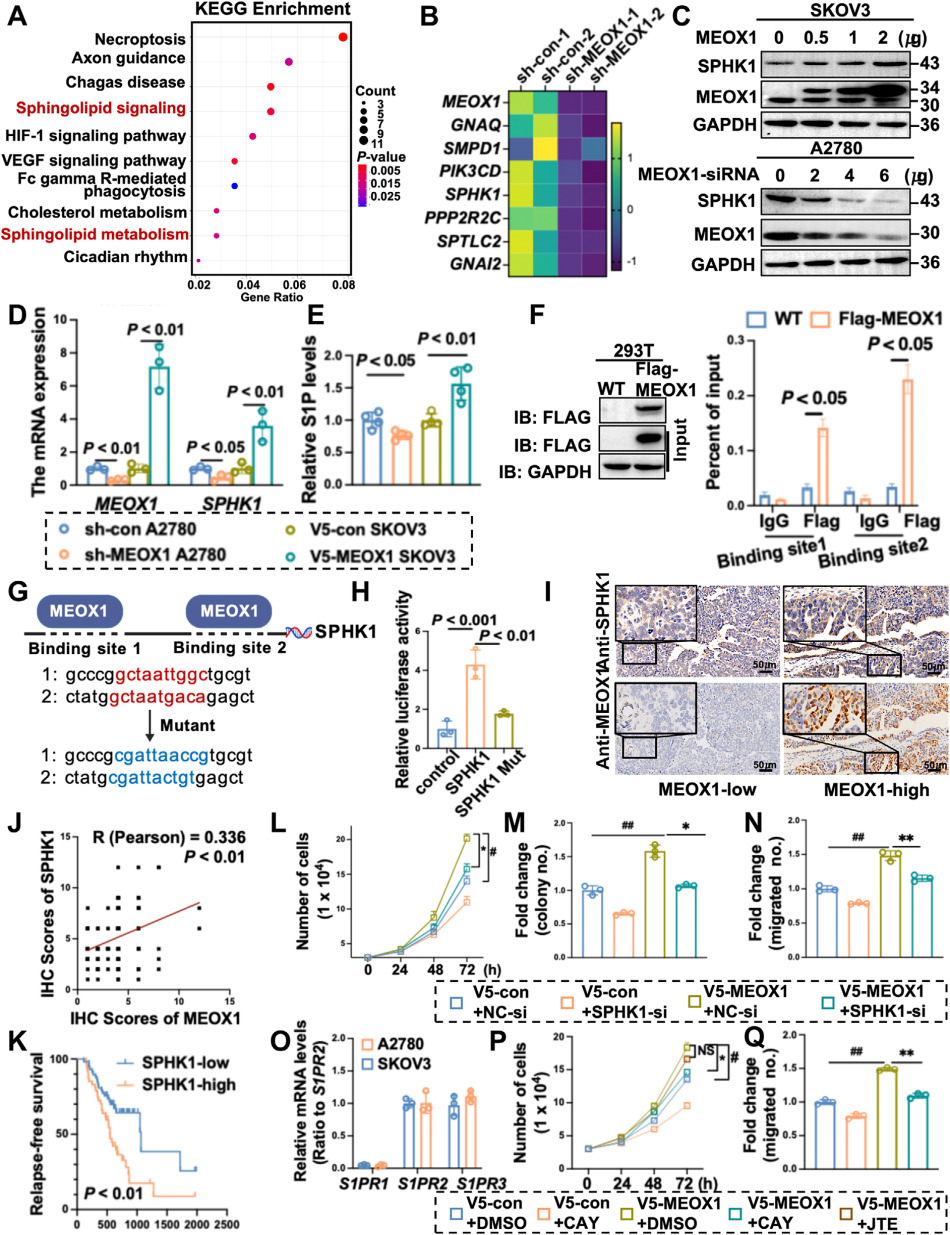
MEOX1 upregulated the SPHK1/S1P axis to enhance ovarian cancer cell proliferation and migration. (A) KEGG pathway enrichment analysis of the downregulated DEGs in sh‐MEOX1 A2780 cells versus sh‐con A2780 cells. (B) The heatmap of DEGs within the sphingolipid signaling pathway. (C) Western blot analysis of MEOX1 and SPHK1 protein levels in SKOV3 cells transfected with increasing amounts of V5‐MEOX1 plasmid or empty vector control (top) and A2780 cells transfected with indicated concentrations of MEOX1‐targeting or control siRNA (bottom). GAPDH served as a loading control. Transfection duration: 72 h. (D) RT‐qPCR analysis of *MEOX1* and *SPHK1* mRNA levels in A2780 cells transfected with LV‐MEOX1‐shRNA (knockdown) and SKOV3 cells transfected with LV‐MEOX1 (overexpression) for 48 h, compared with their respective controls. (E) S1P concentrations in culture supernatants were measured by ELISA in A2780 cells transfected with LV‐MEOX1‐shRNA and SKOV3 cells transfected with LV‐MEOX1 for 48 h. (F) ChIP‐qPCR analysis in 293T cells transfected with Flag‐tagged MEOX1 overexpression plasmid (Flag‐MEOX1 group) or the control plasmid (WT group), showing enrichment of MEOX1 at two predicted binding sites (Site 1: gctaattggc (+818‐79); Site 2: gctaatgaca (+827−88)) in the *SPHK1* promoter. IgG was used as a negative control. (G) Schematic diagram of the wild‐type and mutated MEOX1‐binding site within the *SPHK1* promoter region. (H) Relative luciferase activity was measured in SKOV3 cells co‐transfected with an MEOX1 plasmid, the pRL‐TK Renilla luciferase internal control, and one of the following firefly luciferase reporters: the pGL3‐Basic empty vector (control), pGL3‐SPHK1 promoter (SPHK1), or pGL3‐SPHK1 Mutant promoter (SPHK1 Mut). Firefly luciferase activity was normalized to Renilla luciferase activity for each sample 24 h post‐transfection. (I,J) Representative IHC staining images (I) and correlation analysis (J) between SPHK1 and MEOX1 IHC scores in 113 OC samples (Pearson analysis). (K) RFS analysis was performed in a cohort of 104 OC patients with complete follow‐up information stratified by SPHK1 IHC expression levels (SPHK1‐high vs. SPHK1‐low, SPHK1‐high: IHC score ≥ 6; SPHK1‐low: IHC score < 6). The log‐rank test revealed a statistically significant difference between the two survival curves. (L) Cell Counting assays were performed to assess the proliferation of V5‐con and V5‐MEOX1 SKOV3 cells transfected with NC‐siRNA or SPHK1‐siRNA. Cell viability was measured at 0‐, 24‐, 48‐, and 72‐hours post‐transfection. (M,N) Quantification of colony formation assays (M) and Transwell migration (N) assays. Both assays were performed using V5‐con and V5‐MEOX1 SKOV3 cells transfected with NC‐siRNA or SPHK1‐siRNA for 48 h. (O) RT‐qPCR assays were used to detect the mRNA levels of *S1PR1‐3* in A2780 and SKOV3 cells. (P) Cell counting assays. V5‐con SKOV3 cells or V5‐MEOX1 SKOV3 cells were treated with or without the S1PR2‐specific inhibitor (JTE‐013, 20 µm) or the S1PR3‐specific inhibitor (CAY10444, 20 µm), and then the cell proliferation ability was measured at different time points. (Q) Transwell migration assays. Quantification of the number of migrated cells of V5‐con or V5‐MEOX1 SKOV3 cells treated with or without 20 µm CAY10444 for 24 h. Bar graphs are shown as mean ± SD from three or four independent experiments. NS, no statistical difference; *p < 0.05, **p < 0.01, #*p *< 0.05, ##*p *< 0.01.

To elucidate the downstream molecular mechanism of MEOX1 in driving LNM of OC, RNA sequencing (RNA‐seq) was performed on MEOX1‐knockdown A2780 cells (sh‐MEOX1) and the control cells (sh‐con). Our results revealed 101 downregulated and 122 upregulated DEGs in sh‐MEOX1 A2780 cells compared with sh‐con A2780 cells (|log2FC| > 1, *p*‐value < 0.05, Figure S7A). Kyoto Encyclopaedia of Genes and Genomes (KEGG) pathway enrichment analysis indicated that the significantly downregulated DEGs were mainly enriched in the sphingolipids signaling pathway (Figure 4A), with sphingosine kinase 1 (*SPHK1*) exhibiting the highest fold change (log2FC = −1.77, sh‐MEOX1 vs. sh‐con) and a relatively high expression abundance (Figure 4B; Figure S7B).

Because SPHK1, which catalyzes the phosphorylation of sphingosine to sphingosine‐1‐phosphate (S1P)—a lipid messenger orchestrating both autocrine and paracrine signaling in cancer—represents the key enzymatic node of this pathway, we then examined whether MEOX1 directly regulates its transcription [41, 42]. Our findings showed that MEOX1 knockdown in A2780 cells significantly inhibited the mRNA and protein expression of SPHK1, accompanied by decreased secretion of S1P; Conversely, MEOX1 overexpression in SKOV3 cells upregulated SPHK1 expression and increased S1P production (Figure 4C–E). Chromatin‐immunoprecipitation (ChIP)‐qPCR analysis revealed significant enrichment of MEOX1 at two predicted binding sites within the *SPHK1* promoter in Flag‐MEOX1 plasmids transfected into 293T cells (Figure 4F). To determine whether MEOX1 directly regulates the transcriptional activity of *SPHK1*, a dual‐luciferase reporter assay in SKOV3 cells was performed. Our results showed a significant increase in *SPHK1* promoter activity following MEOX1 overexpression, whereas mutation of the binding motif abolished this effect (Figure 4G,H), suggesting that MEOX1 directly binds to and transcriptionally activates the *SPHK1* promoter at a specific *cis‐*regulatory element. Moreover, we observed a positive correlation between SPHK1 and MEOX1 expression in human OC tissue samples (Figure 4I,J), and worse clinical outcomes (reduced RFS, OS, and PFS) in patients with high SPHK1 expression (Figure 4K; Figure S8).

LNM arises from tumor cell‐intrinsic adaptations, particularly EMT, and dysregulated survival pathways, which collectively facilitate metastatic dissemination [43]. Building on evidence that S1P regulates key oncogenic processes, including cell survival, proliferation, and migration through autocrine signaling [44], we investigated whether the MEOX1/SPHK1/S1P axis influences the malignant behaviors of OC cells. Using SPHK1‐targeting siRNA, we successfully knocked down SPHK1 expression in V5‐MEOX1 and V5‐con‐transfected SKOV3 cells (Figure S9A). Functional assays demonstrated that MEOX1 overexpression significantly enhanced OC cell proliferation (Figure 4L), colony formation (Figure 4M; Figure S9B), and migration (Figure 4N; Figure S9B), while all these pro‐tumorigenic effects were markedly attenuated by SPHK1 inhibition. These results established SPHK1 as a critical downstream effector of MEOX1 in promoting aggressive tumor behaviors. S1P is secreted into the extracellular milieu, where it activates G protein‐coupled S1P receptors (S1PRs) to mediate diverse pathophysiological processes, including fibrosis, tumor progression, metabolic disorders, and vascular dysfunction [44]. Of the five known S1PR subtypes (S1PR1‐5), S1PR1‐3 are ubiquitously expressed, S1PR4 is predominantly localized in lymphoid tissues, and S1PR5 is restricted to the brain and spleen [45]. To determine which S1PRs mediate S1P's autocrine effects in OC, we first analyzed receptor expression patterns. RT‐qPCR revealed that *S1PR2* and *S1PR3* mRNA levels were markedly higher than *S1PR1* levels in both SKOV3 and A2780 cells (Figure 4O). Functional studies demonstrated that the S1PR3‐specific inhibitor CAY10444, but not the S1PR2‐specific inhibitor JTE‐013, significantly suppressed the proliferation of MEOX1‐overexpressing SKOV3 cells (Figure 4P). Consistent with this finding, CAY10444 treatment abolished the enhanced migration capacity of SKOV3 cells induced by MEOX1 overexpression (Figure 4Q; Figure S9C). Transcriptomic data (GEPIA 2.0) showed that *S1PR3* was uniquely overexpressed in OC tissues compared with normal ovaries (Figure S10A). Moreover, high *S1PR3* expression was correlated with worse survival in the Kaplan–Meier Plotter database (Figure S10B). Collectively, these findings indicate that MEOX1 is a key transcriptional regulator of the SPHK1/S1P axis, forming a transcription‐lipid signaling axis that facilitates ovarian cancer progression.

### 2.5 The MEOX1‐SPHK1/S1P Axis Reprograms Fibroblasts Toward a CAF‐Like, Contractile Phenotype

Normal fibroblasts (NFs) can acquire CAF‐like features under tumor‐derived stimuli, characterized by α‐SMA, fibroblast activation protein (FAP), vimentin, and PDGF receptor β (PDGFRB) expression and enhanced matrix remodeling capacity [11, 37, 46, 47]. Once activated, fibroblast functions such as proliferation, migration, secretion of soluble factors, synthesis of ECM components, and ECM regulatory factors (such as MMPs) are enhanced [11, 48]. Given that SPHK1/S1P signaling has been implicated in fibroblast activation and fibrotic responses [49, 50, 51, 52, 53], we next examined whether MEOX1‐induced SPHK1/S1P activation drives fibroblast recruitment and activation in ovarian cancer.

**FIGURE 5:**
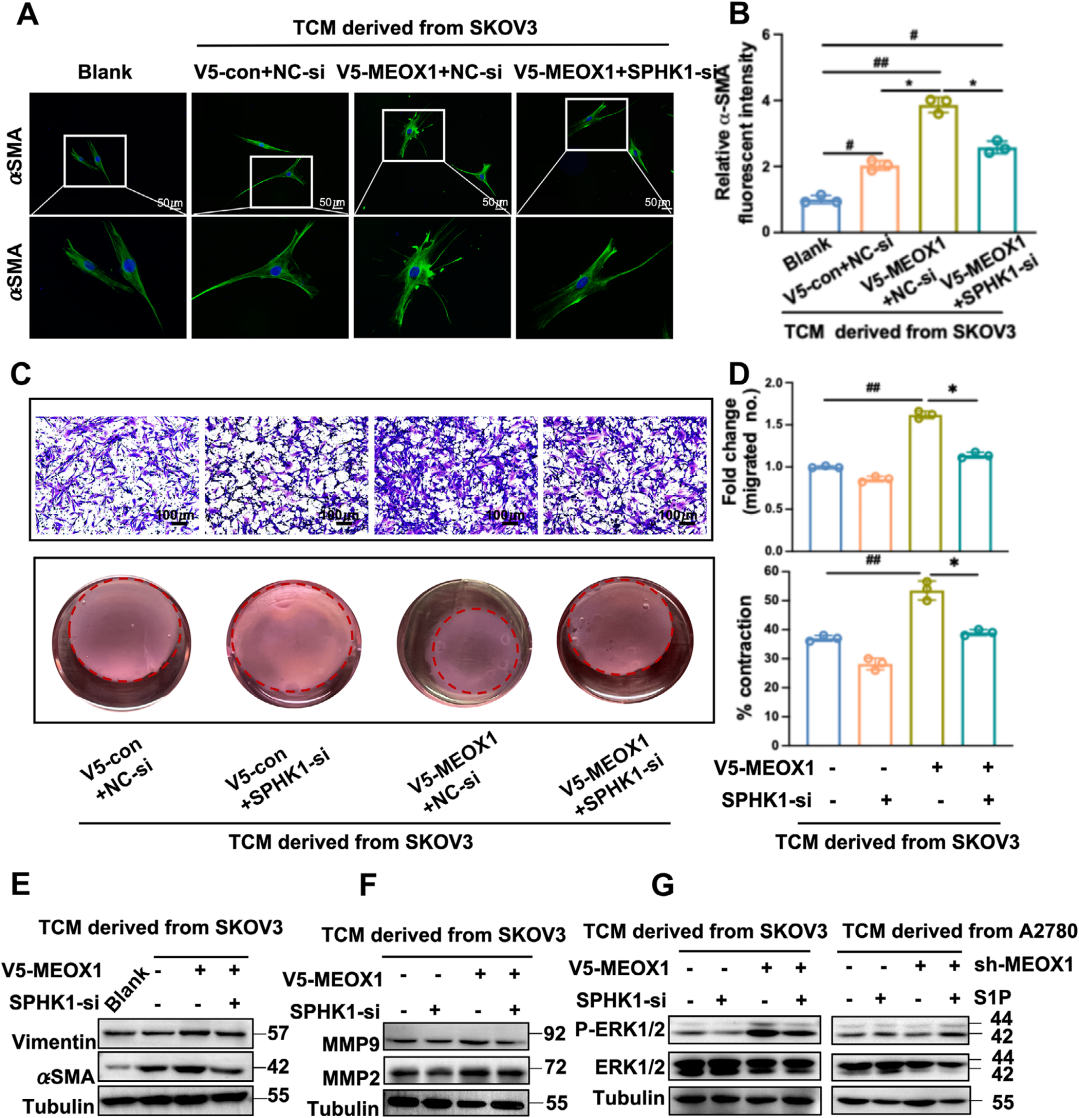
MEOX1/SPHK1/S1P signaling pathway of ovarian cancer cells promoted the activation of fibroblasts. (A) Representative cellular immunofluorescence images showing cell morphology and α‐SMA protein (green) in NFs cultured for 5 days with TCM from V5‐con or V5‐MEOX1 SKOV3 cells transfected with SPHK1‐siRNA or NC‐siRNA. Nuclei were stained with DAPI (blue). Merged channels are shown. (B) Quantitative analysis of α‐SMA fluorescence intensity. (C) Transwell migration assays and collagen gel contraction assays. Representative staining images of NFs that migrated (*upper*) and representative images of collagen gels containing NFs (lower) after treatment for 5 days with different TCM from V5‐con or V5‐MEOX1 SKOV3 cells transfected with SPHK1‐siRNA or NC‐siRNA. (D) Quantitative analysis of the number of migrated NFs and collagen contraction rate, which was calculated as (gel area/ well area) × 100%. (E,F) Western blot assays were used to detect the expression of α‐SMA protein, vimentin protein (E), and MMP2/9 protein (F) in NFs under TCM from V5‐con or V5‐MEOX1 SKOV3 cells transfected with SPHK1‐siRNA or NC‐siRNA. (G) Western blot analyses were performed to detect the expression of P‐ERK1/2 and ERK1/2 proteins in NFs treated with different TCM from V5‐con or V5‐MEOX1 SKOV3 cells transfected with SPHK1‐siRNA or NC‐siRNA (left), or in NFs treated with sh‐con/sh‐MEOX1 TCM with or without 0.1 µm S1P (*right*). Bar graphs are shown as mean ± SD from three independent experiments. *p < 0.05, #*p *< 0.05, ##*p *< 0.01.

Consistent with the association between MEOX1 and CAF markers, bioinformatic analyses of the GEPIA and TIMER2.0 databases revealed strong positive correlations between *SPHK1* mRNA expression and fibroblast activation markers (*FAP*, *ACTA2*, *PDGFRB*, and *S100A4*), as well as higher CAF infiltration scores in OC tissues (Figure S5A,B). These findings suggested that MEOX1/SPHK1 signaling might mediate tumor–stroma communication and fibroblast activation in the OC microenvironment. To experimentally validate these associations, human NFs were treated with TCM derived from V5‐MEOX1 or V5‐con SKOV3 cells. Compared with the blank group (untreated fibroblasts), TCM exposure induced characteristic fibroblast activation, marked by irregular morphology with shortened, widened cell bodies and increased protrusions, alongside significantly elevated α‐SMA expression (Figure 5A,B). These pro‐fibrotic changes were markedly enhanced by MEOX1‐overexpressing TCM compared to control but were largely abolished when SPHK1 was knocked down in donor cells (Figure 5A,B), indicating that fibroblast activation is dependent on MEOX1‐mediated SPHK1 expression.

Functionally, MEOX1‐derived TCM increased fibroblast migration and collagen–gel contraction, and both effects were reversed by SPHK1 knockdown (Figure 5C,D), confirming that SPHK1 activity is indispensable for MEOX1‐driven fibroblast activation. Western blot analysis further demonstrated that fibroblasts exposed to MEOX1‐high TCM expressed higher levels of vimentin, α‐SMA, and the matrix‐remodeling enzymes MMP2 and MMP9, all of which were reduced upon SPHK1 silencing (Figure 5E,F). The ERK pathway has been established as a critical mediator of fibroblast activation in both fibrotic diseases and tumor progression [54, 55, 56]. Previous studies have shown that SPHK1/S1P signaling promotes malignant behavior in various cancers, including OC, by enhancing ERK1/2 phosphorylation [57, 58]. At the signaling level, fibroblasts treated with MEOX1‐high TCM displayed robust ERK1/2 phosphorylation, while this activation was suppressed by SPHK1 knockdown (Figure 5G). Conversely, addition of exogenous S1P restored ERK1/2 phosphorylation in fibroblasts cultured with TCM from MEOX1‐silenced A2780 cells (Figure 5G), confirming that the S1P‐ERK axis operates downstream of MEOX1/SPHK1 signaling.

Together, these findings demonstrate that MEOX1 upregulation in ovarian cancer cells activates SPHK1‐dependent S1P secretion, which reprograms fibroblasts into a contractile, ECM‐remodeling CAF‐like phenotype through ERK pathway activation. Thus, MEOX1 endows tumor cells with the ability to reprogram the stroma, demonstrating that metastatic success requires coordinated enhancement of both tumor fitness and microenvironment engineering.

### 2.6 OC Cell Intrinsic MEOX1 Drives Lymphangiogenesis Through Stromal Reprogramming via the MEOX1/SPHK1/S1P Axis

**FIGURE 6:**
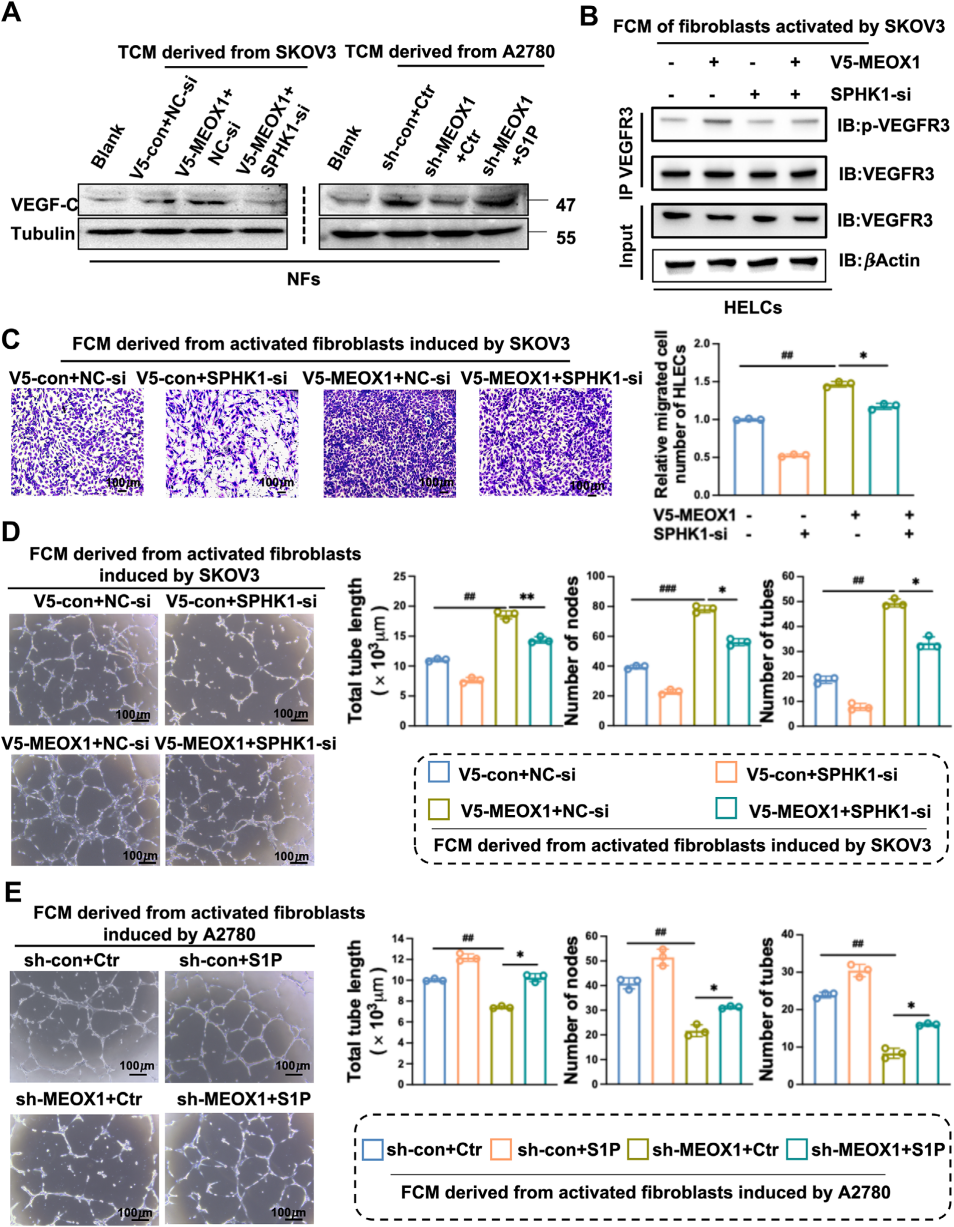
MEOX1/SPHK1/S1P signaling pathway in ovarian cancer cells promoted HLEC migration and tube formation by activating fibroblasts. (A) Western blot assays were conducted to determine the protein level of VEGF‐C in NFs treated with TCM from V5‐con or V5‐MEOX1 SKOV3 cells transfected with SPHK1‐siRNA or NC‐siRNA, or in NFs treated with TCM from sh‐con or sh‐MEOX1 A2780 cells with or without 0.1 µm S1P. (B) Immunoprecipitation assays. HLECs were co‐cultured with NFs pre‐activated by TCM from V5‐con or V5‐MEOX1 SKOV3, with or without SPHK1 knockdown. Following co‐culture, HLEC lysates were immunoprecipitated (IP) with an anti‐VEGFR3 antibody, and the immunoprecipitates were immunoblotted (IB) with a pan‐phospho‐tyrosine antibody (for p‐VEGFR3) and an anti‐VEGFR3 antibody (for total VEGFR3). Whole cell lysates (Input) were immunoblotted for VEGFR3 and 𝛽‐Actin. (C) Transwell migration assays. Left: Representative staining images showing HLECs that migrated after treatment for 24 h with FCMs from activated fibroblasts induced by V5‐con or V5‐MEOX1 SKOV3 cells transfected with SPHK1‐siRNA or NC‐siRNA. Right: Quantitative analysis of the number of migrated HLECs. (D) Representative images of tube formation treated by HLECs for 24 h with FCMs from activated fibroblasts induced by V5‐con or V5‐MEOX1 SKOV3 cells transfected with SPHK1‐siRNA or NC‐siRNA (left) and quantification of total tube length, the number of nodes, and the number of tubes (*right*). (E) Representative images of HLEC tube formation after treatment with FCM derived from activated fibroblasts induced by TCM from A2780 sh‐con or sh‐MEOX1 cells, with or without 0.1 µm S1P (left) and quantification of total tube length, the number of nodes, and the number of tubes (*right*). Bar graphs are presented as mean ± SD from three independent experiments. NS, no statistical difference; *p < 0.05, ##*p *< 0.01, ###*p *< 0.001.

Based on our finding that MEOX1 activates CAFs via the SPHK1/S1P axis, we next investigated the downstream signaling and effector mediating this tumor–stroma crosstalk in lymphangiogenesis. As shown in Figure S11, S1P treatment robustly induced multiple pro‐lymphangiogenic factors in fibroblasts, with VEGF‐C showing the most substantial upregulation. TCM from MEOX1‐overexpressing or ‐silenced OC cells correspondingly upregulated or downregulated VEGF‐C expression in fibroblasts at both transcriptional (Figure S12A) and protein (Figure 6A) levels in a SPHK1/S1P‐dependent manner. Mechanistically, the finding that MEOX1‐driven fibroblast activation induced robust ERK1/2 phosphorylation through SPHK1/S1P signaling (Figure 5G) prompted us to investigate its mediatory role. Indeed, ERK inhibition with SCH772984 partially abrogated the MEOX1‐induced upregulation of VEGF‐C in fibroblasts at both mRNA and protein levels (Figure S12B,C), indicating that ERK signaling is essential for coupling the MEOX1/SPHK1/S1P axis to VEGF‐C secretion in CAFs.

Having identified VEGF‐C as the key paracrine effector downstream of this axis, we sought to determine whether VEGF‐C from MEOX1/SPHK1‐activated fibroblasts directly acts on HLECs. Co‐culture of HLECs with NFs pre‐activated by TCM from SKOV3 cells revealed that MEOX1‐overexpressing tumor cells markedly increased HLEC VEGFR3 phosphorylation, an effect abolished by SPHK1 knockdown, without altering total VEGFR3 levels (Figure 6B). To functionally validate this paracrine mechanism, NFs were exposed to tumor‐conditioned medium to generate activated fibroblasts, then activated fibroblast‐conditioned medium (FCM) was used to treat the HLECs. FCM of fibroblasts activated by MEOX1‐overexpressing OC cells significantly enhanced HLEC migration compared with control FCM, an effect abolished by SPHK1 knockdown in tumor cells (Figure 6C). Similarly, FCM from fibroblasts activated by MEOX1‐overexpressing OC cells markedly promoted HLEC tube formation, whereas FCM from fibroblasts activated by MEOX1‐knockdown cells inhibited this process (Figure 6D,E). Importantly, these effects were significantly attenuated by SPHK1 inhibition in V5‐MEOX1 SKOV3 cells (Figure 6D) and by S1P supplementation in sh‐MEOX1 TCM, respectively (Figure 6E). These findings confirm that MEOX1/SPHK1‐activated fibroblasts directly stimulate HLEC activation and lymphangiogenesis through paracrine VEGF‐C.

### 2.7 The MEOX1/SPHK1/S1P/S1PR1 Axis Drives CAF‐Mediated Lymphangiogenesis and Lymph Node Metastasis in Ovarian Cancer

**FIGURE 7:**
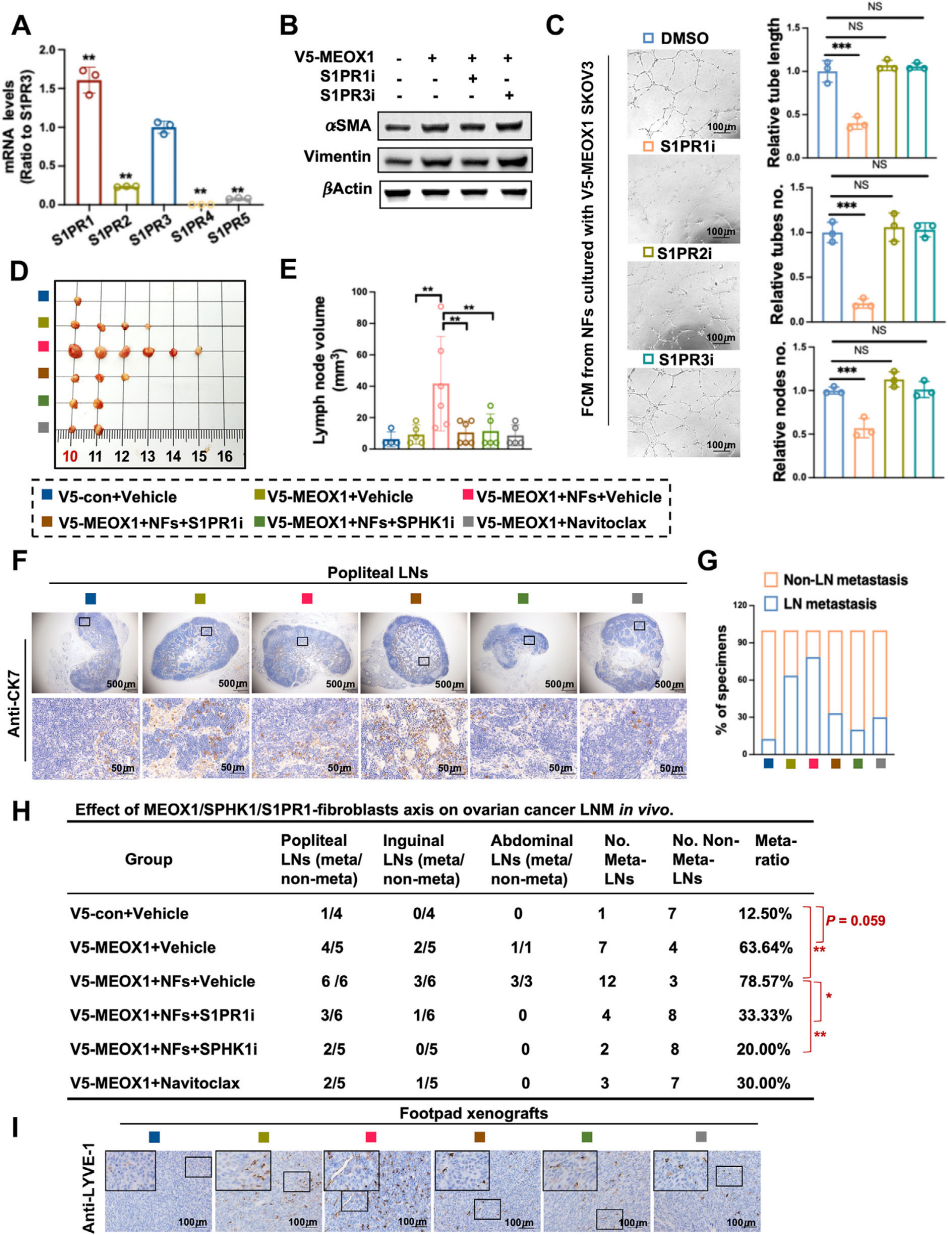
The MEOX1/SPHK1/S1PR1 axis activates fibroblasts to promote lymphangiogenesis and lymph node metastasis in ovarian cancer. (A) RT‐qPCR assays were performed to detect mRNA levels of *S1PR1‐5* in primary CAFs. Data are presented as mean ± SD from three independent experiments. **p < 0.01 versus *S1PR3*. (B) Western blot assays were conducted to determine the protein levels of α‐SMA and Vimentin in NFs treated with TCM from V5‐MEOX1 SKOV3 cells in the presence of 20 µm S1PR1 inhibitor (S1PR1i, W146) or 20 µm S1PR3 inhibitor (S1PR3i, CAY10444). β‐Actin served as a loading control. (C) Tube formation assay of HLECs treated with FCM from NFs activated as in (B) in the presence of 20 µm S1PR1i, S1PR2 inhibitor (S1PR2i, JTE‐013), or S1PR3i. Scale bar, 100 µm. Representative images of HLEC tube formation (left) and quantification of total tube length, tube number, and node number (*right*). Bar graphs are presented as mean ± SD from three independent experiments. NS, no statistical difference; ***p < 0.001. (D) Representative images of swollen popliteal lymph nodes (diameter > 2 mm) harvested from mice bearing footpad xenografts of the indicated groups (*n* = 6): V5‐con+Vehicle, V5‐MEOX1+Vehicle, V5‐MEOX1+NFs+Vehicle, V5‐MEOX1+NFs+S1PR1i, V5‐MEOX1+NFs+SPHK1i, and V5‐MEOX1+Navitoclax. (E) Quantification of lymph node volume from each group. Data are presented as mean ± SD. **p < 0.01. (F) Representative anti‐CK7 IHC staining of popliteal lymph nodes sections. Upper panels: scale bar, 500 µm; lower panels: scale bar, 50 µm. (G) Quantification of lymph node metastasis incidence across popliteal, inguinal, and abdominal lymph nodes. Data are shown as the percentage of metastatic lymph nodes relative to total lymph nodes examined. (H) Summary table of lymph node metastasis of each group. Meta ratio is defined as the percentage of metastatic LNs relative to total LNs examined. *p < 0.05, **p < 0.01 (Fisher's exact test). (I) Representative anti‐LYVE‐1 IHC staining of footpad xenograft sections of each group. Scale bar, 100 µm.

To identify the S1P receptors responsible for transducing the pro‐activation and pro‐lymphangiogenic effects of MEOX1/SPHK1‐derived S1P on fibroblasts, we first profiled *S1PR1‐5* expression in primary CAFs. Among the five receptors, *S1PR1* and *S1PR3* showed the highest mRNA levels (Figure 7A). Subsequent functional analyses​ using subtype‐selective inhibitors revealed that pharmacological blockade of S1PR1 (S1PR1i), but not S1PR3 (S1PR3i), suppressed MEOX1‐induced NF activation, as evidenced by reduced expression of the activation markers α‐SMA and Vimentin (Figure 7B). Furthermore, S1PR1 blockade, but not S1PR2 or S1PR3 inhibition, abolished the enhanced tube formation capacity of HLECs induced by MEOX1‐activated fibroblasts (Figure 7C). These results identify S1PR1 as the predominant receptor mediating S1P‐induced fibroblast activation and subsequent lymphangiogenesis.

To causally link the MEOX1/SPHK1/S1P/S1PR1 signaling to fibroblast activation and subsequent lymphangiogenesis in vivo, we performed rescue experiments using a mouse popliteal lymph node metastasis model. Results showed that MEOX1 overexpression alone (V5‐MEOX1+Vehicle) significantly elevated the total LNM rate to 63.64% compared with 12.50% in the control group (*p* = 0.059), with metastases extending to inguinal and abdominal lymph nodes (Figure 7G,H). In line with the previous animal cohort (Figure 2H), normalization of the metastatic index showed that MEOX1‐overexpressing tumors displayed a significant higher LNM index than controls (*p* < 0.05, Figure S13), further supporting that the pro‐metastatic effect of MEOX1 on LNM may not be solely attributable to enhanced primary tumor growth. Co‐injection of NFs with MEOX1‐overexpressing cells (V5‐MEOX1+NFs+Vehicle) further exacerbated metastatic burden, yielding the largest popliteal lymph node volume (Figure 7D and 7E) and the highest metastasis rate (78.57%, Figure 7G,H), indicating that CAFs may serve as critical mediators of LNM. Strikingly, compared to the V5‐MEOX1+NFs+Vehicle group, treatment with either the S1PR1 inhibitor (S1PR1i) or the SPHK1 inhibitor (SPHK1i) markedly reduced popliteal lymph node volume (*p* < 0.01, Figure 7D,E) and decreased the metastasis rate to 33.33% (*p* < 0.05) and 20% (*p* < 0.01), respectively (Figure 7G,H). Similarly, CAF depletion using Navitoclax [59, 60], reduced the metastasis rate to 30.00%, compared with 63.64% in the V5‐MEOX1 alone group. Consistent with the LNM data, the V5‐MEOX1+NFs+Vehicle group appeared to exhibit the highest lymphatic vessel density, whereas inhibition of S1PR1, SPHK1, or CAF depletion showed a tendency toward reduced lymphatic vessel density (Figure 7I). These in vivo findings demonstrate that the MEOX1/SPHK1/S1PR1 axis is functionally required for CAF‐driven lymphangiogenesis and lymphatic metastasis, and that CAFs play an indispensable role in this process.

**FIGURE 8:**
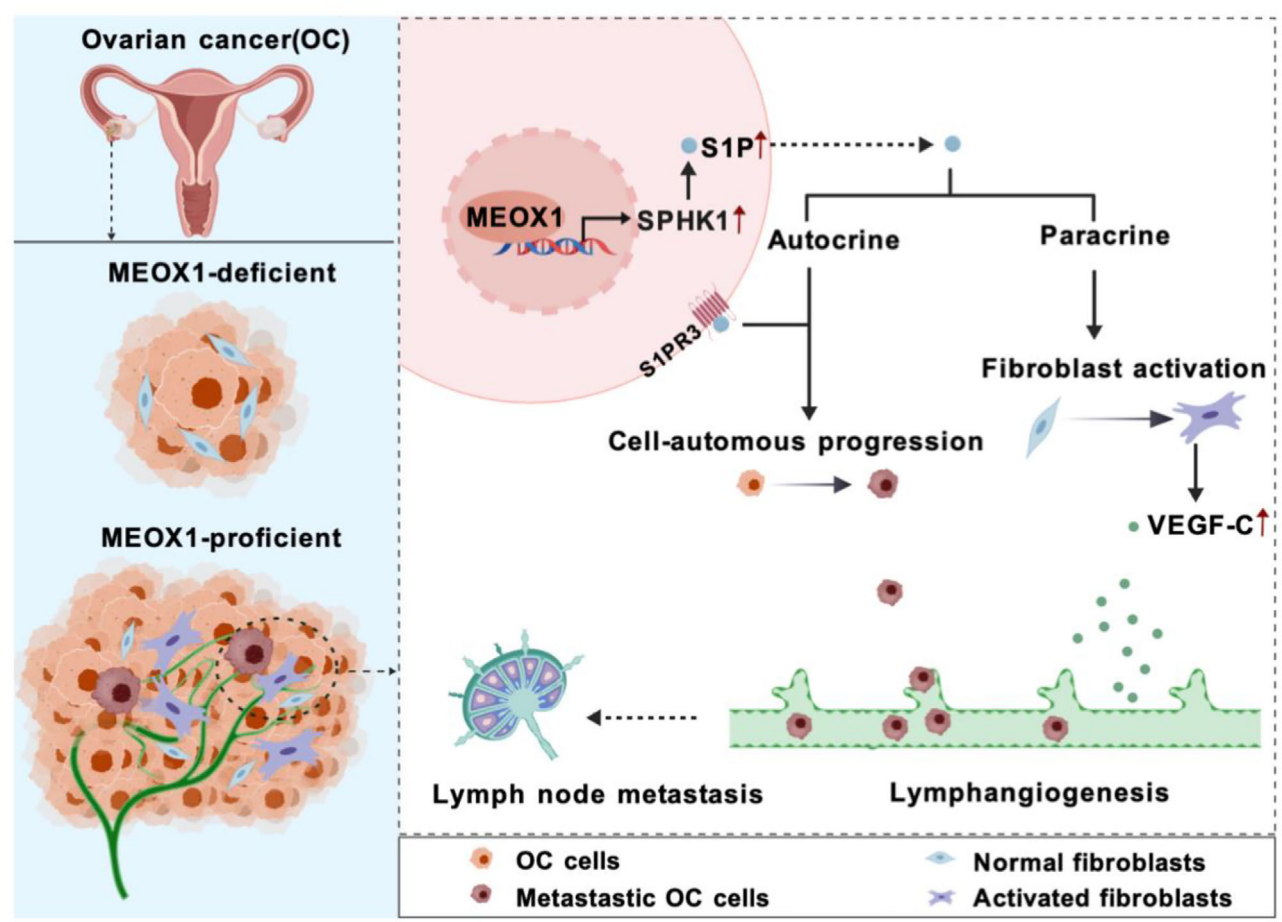
Proposed model of MEOX1‐induced fibroblast activation via SPHK1/S1P axis to promote ovarian cancer lymphangiogenesis and LNM. MEOX1 upregulates SPHK1 expression in ovarian cancer cells, leading to increased production and secretion of S1P. S1P acts not only in an autocrine manner, via S1PR3 to enhance tumor cell proliferation and migration, but also in a paracrine fashion, activating normal fibroblasts into CAF‐like status that secrete VEGF‐C. This VEGF‐C‐rich microenvironment subsequently stimulates migration and tube formation of LECs, promotes lymphangiogenesis, and ultimately facilitates LNM in OC. This schematic was created with BioGDP.com [61].

In summary, we demonstrate that tumor cell‐intrinsic MEOX1 transcriptionally activates SPHK1/S1P to promote LNM through a dual mechanism: (1) autocrine SPHK1/S1P/S1PR3 signaling that enhances tumor cell proliferation and migration, and (2) paracrine activation of fibroblasts into a pro‐metastatic CAF‐like phenotype that drives VEGF‐C‐dependent lymphangiogenesis (Figure 8). These findings establish MEOX1 as a central node coordinating both tumor‐autonomous and microenvironmental reprogramming in ovarian cancer metastasis.

## 3 Discussion

LNM is a primary determinant of poor survival in solid tumors such as OC [1, 2], though the underlying mechanisms orchestrating this process remain incompletely understood. Our findings support a general principle of metastasis: tumor cells must not only acquire intrinsic malignant traits but also actively sculpt a supportive stromal and lymphatic niche to enable metastatic escape. Through integrated clinical, spatial, molecular, and functional evidence, we identify MEOX1 as a key orchestrator of this dual requirement. Our data unveil a previously unrecognized autocrine‐paracrine switch: by transcriptionally activating SPHK1, MEOX1 initiates a dual‐signaling output. The resulting S1P pool simultaneously fuels tumor cell proliferation/migration via S1PR3 and reprograms fibroblasts into pro‐lymphangiogenic CAFs via S1PR1. This model effectively explains the critical role of the stroma in MEOX1‐driven metastasis, reconciling the discrepancy between its in vivo and in vitro phenotypes.

The functional importance of CAFs in metastatic progression is supported by accumulating preclinical evidence across solid tumors [12, 14, 62, 63]. Moreover, tumor cells in multiple malignancies, including intrahepatic cholangiocarcinoma [59], breast cancer [64], ovarian cancer [16], and bladder cancer [13], were reported to induce fibroblast activation and facilitate lymphangiogenesis and LNM. Consistent with observations in other cancer types [8, 12, 13, 14], our analyses of clinical OC specimens and public spatial transcriptomic data revealed significant spatial proximity between CAFs and HLECs and coordinated enrichment of CAFs and LECs in MEOX1‐high tumors. Furthermore, our data from both patient‐derived OC samples and re‐analyzed public scRNA‐seq cohorts demonstrated CAFs as the predominant source of VEGF‐C, outperforming tumor cells in VEGF‐C production, which is consistent with previous reports [65, 66]. Crucially, we resolved the apparent paradox between MEOX1's strong pro‐lymphatic metastatic effects in vivo and its inability to directly stimulate LEC tubulogenesis in vitro by identifying CAF‐mediated signaling as the essential intermediary. Functional characterization confirmed that MEOX1‐expressing tumor cells activate normal fibroblasts, inducing characteristic CAF markers (α‐SMA, FAP) and enhancing ECM remodeling capacity.

Mechanistically, we demonstrate that MEOX1 integrates transcriptional regulation to lipid metabolism by directly binding to the *SPHK1* promoter, ensuring a sustained S1P production. The SPHK1/S1P axis is a well‐established regulator of cancer progression, influencing cell survival, migration, angiogenesis, chemotherapy resistance, cancer stemness, and stromal crosstalk across malignancies [67, 68, 69, 70, 71, 72]. In OC, it has been linked to hypoxia response and adipocyte‐induced proliferation [58, 73]. Both SPHK1/S1P and MEOX1 have been implicated in fibroblast activation under pathological conditions such as cardiac dysfunction, pulmonary fibrosis, corneal fibrosis, and renal fibrosis [49, 50, 51, 52, 53]. The clinical relevance of this pathway is underscored by the significant correlation between MEOX1 and SPHK1 expression in human OC samples, as well as the association between *MEOX1/SPHK1* mRNA levels and CAF infiltration and activation. Our functional experiments delineate a linear pathway whereby MEOX1 acts through the SPHK1/S1P axis to activate fibroblasts, and this signal is transduced specifically via S1PR1, ultimately driving CAF‐mediated lymphangiogenesis.

Consistent with this model, we found that MEOX1/SPHK1/S1P‑activated fibroblasts secrete abundant VEGF‑C and induce VEGFR3 phosphorylation on LECs in a paracrine manner, thereby promoting LEC tube formation. This finding aligns with the established role of VEGF‐C as a master regulator of lymphangiogenesis and positions CAFs as a major cellular source of this factor in the OC tumor microenvironment. Importantly, our in vivo rescue experiments provide causal evidence for this paracrine axis. MEOX1 overexpression significantly enhanced LNM and stimulated lymphangiogenesis in xenograft tumors, effects that were synergistically potentiated by co‐injection of NFs and, notably, abrogated by pharmacological inhibition of S1PR1 or SPHK1, phenocopying CAF depletion. These observations collectively demonstrate that CAFs are not merely passive bystanders but rather functional enablers of MEOX1‐driven metastasis, highlighting the therapeutic potential of disrupting this tumor–stroma crosstalk.

While MEOX1 has been previously associated with LNM in breast and lung cancers [27, 31], those studies were confined to correlative immunohistochemical analyses with no functional interrogation. Our own prior work in ovarian cancer [20] similarly fell short of establishing causality or delineating the mechanism. The present study makes several conceptual advances beyond these preliminary findings. First, we provide definitive functional evidence that MEOX1 is a critical mediator​ of lymph node metastasis in ovarian cancer. Second, and more importantly, we identify the SPHK1/S1P axis as a direct downstream effector of MEOX1, which promotes metastasis by activating fibroblasts. To our knowledge, this mechanistic link between MEOX1, lipid signaling, and stromal reprogramming has not been previously reported, representing a fundamental leap from phenotype to mechanism. Additionally, genetic or pharmacological inhibition of SPHK1, S1PR1, or S1PR3 effectively attenuated MEOX1‐induced oncogenic phenotypes and lymphatic metastasis. The consistency across our molecular, spatial, and clinical datasets further underscores the clinical relevance of this pathway. Importantly, given the availability of selective SPHK1 inhibitors and S1PR3 antagonists (e.g., SPHK1 inhibitor PF‐543 and S1PR3 antagonist CAY10444), our study provides a mechanistic and translational rationale for disrupting the MEOX1‐SPHK1/S1P signaling axis as an anti‐lymphangiogenic strategy in ovarian cancer. Furthermore, the relative genomic stability of CAFs further enhances the therapeutic appeal of this approach, potentially mitigating the development of acquired resistance [10, 74]. Future studies should explore nanoparticle‐based delivery systems to selectively target inhibitors of CAFs or LECs, thereby improving their therapeutic indices. Furthermore, S1P levels in ascites or blood [70] could serve as non‐invasive biomarkers for LNM risk stratification.

Several limitations of the present study should be acknowledged. First, we acknowledge that the use of human NFs in this study has certain limitations. NFs differ substantially from primary CAFs derived from OC tumor microenvironment (e.g., CAF precursors or peritumoral fibroblasts) in terms of their basal activation status and responsiveness to signaling cues. Therefore, the regulatory effects of the MEOX1/SPHK1/S1P axis observed in NFs may not fully recapitulate the in vivo situation. Nevertheless, NFs offer a well‐controlled, low‐background system enabling reliable dissection of the core mechanism without pre‐existing tumor‐associated alterations. Accordingly, our NF‐based results serve as a valuable proof‐of‐concept. While we have validated key pro‐lymphangiogenic functions using primary CAFs isolated from OC patients (which yielded results consistent with those from activated NFs), future studies utilizing in vivo models or a broader panel of CAF subtypes will be valuable to fully capture the complexity of the tumor microenvironment. Second, although we have normalized the metastatic index to primary tumor volume to mitigate the potential confounding effect of tumor growth, we acknowledge that this approach does not completely exclude the possibility that the observed increase in LNM upon MEOX1 overexpression. Definitive evidence will require future studies using orthotopic models with carefully matched tumor volumes to dissect the growth‐independent pro‐metastatic function of MEOX1. Third, our findings were primarily supported by in vitro assays and a popliteal LNM model, which incompletely recapitulates ovarian cancer metastasis. Robust in vivo validation in orthotopic or patient‐derived xenograft models and larger‐scale validation across multiple independent cohorts will be necessary to confirm the anti‐lymphangiogenic efficacy and safety of pathway blockade. Last, while VEGF‐C emerged as the dominant effector linking CAF activation to lymphangiogenesis in our system, additional paracrine mediators such as PDGFs, FGFs, or S1P carrier proteins are likely to contribute to niche formation and warrant further investigation.

In summary, by demonstrating how MEOX1 coordinates transcriptional activation, lipid signaling, and stromal remodeling, our study reveals metastasis as a coupled system‐driven not only by tumor‐intrinsic programs but also by active tumor‐mediated niche construction. Through delineating this dual‐arm regulatory circuit, we provide a conceptual and translational framework for understanding lymphangiogenic dissemination in ovarian cancer and highlight the MEOX1‐SPHK1/S1PR3 axis as a promising target for future anti‐lymphatic therapies.

## 4 Materials and Methods

### 4.1 Data Mining for LNM‐Related Genes in Ovarian Cancer

The expression profile data and clinical information of OC were retrieved from the TCGA database (http://portal.gdc.cancer.gov/). Patients were categorized by LNM status into LNM (+) or LNM (−) groups. Using the limma package, LNM‐related DEGs were screened with |log2FC| > 0.585 (equivalent to FC > 1.5 or <1/1.5) and *p*‐value < 0.05. Additionally, four OC gene expression profiles (GSE69428, GSE18520, GSE54388, and GSE27651) were obtained from the GEO database (https://www.ncbi.nlm.nih.gov/geo/). Cancer‐related DEGs were defined as genes consistently differentially expressed (using the same threshold) in at least three of these datasets when comparing OC tissues to normal ovarian tissues. The final set of LNM‐related genes was derived from the intersection between upregulated LNM‐related DEGs from TCGA and upregulated cancer‐related DEGs from the GEO analysis.

### 4.2 Cell Lines and Cell Culture

Human OC cell lines SKOV3 (RRID: CVCL_0532) and A2780 (RRID: CVCL_0134), as well as HEK293T cells (RRID: CVCL_0063Best), were purchased from the American Type Culture Collection (ATCC; Manassas, Virginia, USA) in 2020. HLECs (JY527) were purchased from ScienCell (Carlsbad, CA, USA) in 2021. Primary CAFs and NFs were isolated from ovarian cancer tissues and histologically normal ovarian specimens, respectively, collected from the Obstetrics & Gynecology Hospital of Fudan University with ethical approval (protocol 2021–94). All cell lines were authenticated by short tandem repeat (STR) and confirmed to be free of mycoplasma contamination.

SKOV3 and A2780 cells were cultivated in RPMI‐1640 (SH30809.01, HyClone, Logan, Utah, USA) supplemented with 10% fetal bovine serum (FBS) (10270‐106, GIBCO, Grand Island, NY, USA) and 1 × 10^5^ IU/L penicillin and streptomycin (15140‐122, GIBCO, Grand Island, NY, USA). 293T cells were maintained in DMEM (SH30022.01, HyClone, Logan, Utah, USA) supplemented with 10% FBS and 1 × 10^5^ IU/L penicillin and streptomycin. Primary CAFs and NFs were grown in the fibroblast medium (2301, ScienCell, Carlsbad, CA, USA), while HLECs were cultured in the endothelial cell medium (1001, ScienCell, Carlsbad, CA, USA). All cells were incubated in the cell incubator, maintaining 37°C, 5% CO_2_, and 95% humidity.

### 4.3 The Establishment of MEOX1‐Suppressing or MEOX1‐Overexpressing Stable Cell Lines

Ovarian cancer cells were seeded in 24‐well plates at 2.5 × 10^4^ cells per well. Following attachment, cells were transduced with lentiviral constructs in complete medium for 12 h, including: lentivirus encoding shRNA targeting MEOX1 (LV‐MEOX1‐shRNA, Genechem, Shanghai, China) or its control (LV‐MEOX1‐sh‐con), lentivirus coding MEOX1 (LV‐MEOX1, Genechem, Shanghai, China) or its control (LV‐con), or virus‐free medium (blank control). After 72 h, selection was initiated using puromycin (2.5 µg/mL for SKOV3, 0.8 µg/mL for A2780). Selection continued until complete cell death occurred in blank controls, after which surviving cells were expanded as stable lines.

Specifically, A2780 cells transduced with LV‐MEOX1‐shRNA (target sequence: GGAGGAG‐CACATCTTCACTGA) were designated sh‐MEOX1, with controls as sh‐con. SKOV3 cells transduced with LV‐MEOX1 were designated V5‐MEOX1, with controls as V5‐con.

### 4.4 SiRNA or Plasmid Transfection

Tumor cells were plated into a 6‐well plate and transfected at approximately 60% confluence using jetPRIME (114‐15, Polyplus‐transfection SA, Strasbourg, France) transfection reagent according to the manufacturer's protocol. Briefly, transfection complexes were prepared by incubating 10–50 nm of MEOX1‐ or SPHK1‐ targeting siRNA (or 2 µg of MEOX1 plasmid) with 4 µL jetPRIME reagent in 200 µL jetPRIME Buffer for 10 min at room temperature. The complexes were then added to cells in 2 mL fresh medium. Medium was refreshed according to cell status post‐transfection. The sequences of siRNA (Genechem, Shanghai, China) were as follows: MEOX1‐siRNA1, GAGACAGAGAAGAAAUCAUTT; MEOX1‐siRNA2, GCCCAUCAUAACUACCUG‐ATT; SPHK1‐siRNA1, GCGUCAUGCAUCUGUUCUATT; SPHK1‐siRNA2, GAGGCUGAAA‐UCUCCUUCATT.

### 4.5 Mouse Popliteal Lymph Node Metastasis Model for Ovarian Cancer

Female BALB/c nude mice (approximately 5 weeks old) from Gempharmatech Co., Ltd. (SCXK (Su) 2018‐0008, Nanjing, China) were maintained under SPF‐grade conditions. Mice were randomly divided into two groups (*n* = 8 per group) and received footpad injections of 5 × 10^6^ V5‐MEOX1 or V5‐con SKOV3 cells in 50 µL PBS. Tumor growth and popliteal lymph node swelling were monitored weekly. Thirty days after footpad tumors became macroscopically visible, metastasis was assessed by in vivo fluorescence imaging, followed by euthanasia and collection of footpad tumors and lymph nodes (popliteal, inguinal, abdominal) for measurement and 4% paraformaldehyde fixation.

The in vivo rescue experiments were performed as follows. Female BALB/c nude mice were randomly divided into six groups (*n* = 6 per group): V5‐con+Vehicle, V5‐MEOX1+Vehicle, V5‐MEOX1+NFs+Vehicle, V5‐MEOX1+NFs+S1PR1i, V5‐MEOX1+NFs+SPHK1i, and V5‐MEOX1+Navitoclax. SKOV3 cells (V5‐con or V5‐MEOX1, 5 × 10^6^ cells) were injected alone or together with NFs (5 × 10^5^ cells) into the footpad of each mouse in a total volume of 50 µL PBS. After tumor establishment, mice received the following treatments: S1PR1 inhibitor (W146, MedChemExpress, NJ, USA; 5 mg/kg, intraperitoneal injection, ip), SPHK1 inhibitor (PF‐543, MedChemExpress, NJ, USA; 1 mg/kg, ip), or CAF‐depletion agent (Navitoclax, MedChemExpress, NJ, USA; 100 mg/kg, oral administration, po). The control groups received a vehicle alone. All treatments were administered for 5 weeks. Popliteal, inguinal, and abdominal lymph nodes were harvested, fixed in 4% paraformaldehyde, and paraffin‐embedded for sectioning.

All procedures were approved by the Animal Welfare and Ethics Group, Department of Experimental Animal Science, Fudan University (202209013S).

### 4.6 Clinical Specimens and IHC Staining

A total of 113 OC samples were collected from the Obstetrics & Gynecology Hospital of Fudan University with informed consent and approval from the institute's Ethics Committee (protocol 2021–94). Among these, 104 with complete follow‐up data were included in the RFS analysis. Human OC samples and mouse xenograft samples were fixed, paraffin‐embedded, and sectioned for immunohistochemical analysis.

For IHC staining, slides were deparaffinized, rehydrated (in a gradient series using 100%, 95%, 80%, and 70% ethanol solutions), and subjected to antigen retrieval using Tris‐EDTA buffer (pH 9) (36318ES60, Yeasen, Shanghai, China) after permeabilization with 1% Triton X‐100. After blocking with 5% donkey serum, sections were incubated with primary antibodies at 4°C overnight, followed by HRP‐conjugated secondary antibodies and DAB development (G1212‐200T, Servivebio, Wuhan, China). All slides were counterstained, dehydrated, and mounted for evaluation. IHC scoring was performed by assessing at least three random fields per sample using an Olympus BX53 microscope (Olympus, Tokyo, Japan). The final score (0–12) was calculated by multiplying the percentage score of positive cells (0: 0%; 1: 1%–10%; 2: 11%–50%; 3: 51%–70%; 4: 71%–100%) by the staining intensity score (0: none; 1: weak; 2: moderate; 3: strong). Antibody details are provided in Table S3.

### 4.7 Immunofluorescence Staining

For tissue immunofluorescence, the protocol followed IHC procedures with fluorescently labeled secondary antibodies. After DAPI (D9542, Sigma, St. Louis, MO, USA) nuclear staining, slides were mounted with anti‐fade medium (P0126, Beyotime Biotechnology, Shanghai, China) and imaged using an Olympus BX53 microscope (Olympus, Tokyo, Japan). For cellular immunofluorescence, cells grown on coverslips were fixed with 4% paraformaldehyde (BL539A, Biosharp, Guangzhou, China), permeabilized with 0.5% Triton X‐100, and blocked with donkey serum. Samples were then incubated with primary and fluorescence‐dye‐conjugated secondary antibodies, counterstained with DAPI, anti‐fluorescence quenching, and mounted for imaging under the Olympus BX53 microscope (Olympus, Tokyo, Japan). Antibody details are provided in Table S3.

### 4.8 Multiplex Immunofluorescence Staining

Tissue sections were deparaffinized, rehydrated, subjected to antigen retrieval, and blocked as described for immunofluorescence staining. For multiplex staining, a sequential strategy was employed. Sections were incubated with the primary antibody against the first target overnight at 4°C, followed by HRP‐conjugated secondary antibody for 1 h at room temperature. Signal was developed using tyramide signal amplification with the Opal Multiplex IHC Kit (Akoya Biosciences, Marlborough, MA, USA). To strip the antibody complex, sections were subjected to heat treatment in citrate buffer (pH 6, microwave for 10 min). This process was repeated sequentially for additional targets. Nuclei were counterstained with DAPI. Images were acquired and analyzed as described for immunofluorescence staining. Antibody details are provided in Table S3.

### 4.9 Quantitative Real‐Time Polymerase Chain Reaction (RT‐qPCR)

Total RNA was extracted from cells or tissues using the Total RNA Extraction Reagent (R401‐01, Vazyme, Nanjing, China), followed by cDNA synthesis with the ReverTra Ace qPCR RT Kit (FSQ101, TOYOBO, Osaka, Japan). SYBR Green Realtime PCR Master Mix (QPK‐201, TOYOBO, Osaka, Japan) was used for qPCR. All primer sequences are listed in Table S4.

### 4.10 Western Blotting

Proteins were extracted from cells and quantified using a BCA Protein Assay Kit (P0012, Beyotime Biotechnology, Shanghai, China). After SDS‐PAGE separation and membrane transfer, blots were blocked, incubated with primary and secondary antibodies, and visualized by chemiluminescence (180‐5001, Tanon Science & Technology Ltd, Tanon, Shanghai, China). Antibodies are listed in Table S3.

### 4.11 Cell Counting Assays

V5‐con or V5‐MEOX1 SKOV3 cells (3 × 10^4^/well) were seeded in 48‐well plates following one of two pretreatment conditions: (1) transfection with SPHK1‐siRNA or NC‐siRNA for 24 h; or (2) treatment with 20 µm JTE‐013 (an S1PR2‐specific inhibitor, MedChem Express, Princeton, NJ, USA) or 20 µm CAY10444 (an S1PR3‐specific inhibitor, MedChem Express, Princeton, NJ, USA). Cells in each well were digested and counted at 24‐, 48‐, and 72‐hours post‐seeding.

### 4.12 Colony Formation Assays

Treated cells were implanted in a 6‐well plate at a density of 2000 per well and cultured until colonies became visible to the naked eye. After fixation with 4% paraformaldehyde (BL539A, Biosharp, Guangzhou, China) for 15 min and subsequent staining with 0.5% Crystal Violet Stain Solution (60506ES60, Yeasen, Shanghai, China) for 40 min, the plates were thoroughly rinsed with water and cell colonies (≥10 cells) were counted under a Leica Microsystems CMS GmbH microscope (Leica Microsystems, Buffalo Grove, IL, USA).

### 4.13 Transwell Migration Assays

Treated cells (5–8 × 10^4^/well, in 200 µL complete medium with 10% FBS) were seeded in the upper compartment of the Transwell chamber in a 24‐well plate, and 800 µL complete medium with 20% FBS was added to the lower compartment of the chamber. SKOV3 cells and NFs were incubated for 18 h, while HLECs were cultured for 10 h. The cells were then fixed with 4% paraformaldehyde (BL539A, Biosharp, Guangzhou, China) and stained with 0.5% Crystal Violet Stain Solution (60506ES60, Yeasen, Shanghai, China). Non‐migrating cells in the upper chamber were removed using a cotton swab. The migrated cells on the lower surface were photographed and counted under the Olympus BX53 microscope (Olympus, Tokyo, Japan).

### 4.14 RNA Sequencing and Pathway Enrichment Analysis

Total RNA was extracted from sh‐MEOX1 A2780 cells and sh‐con A2780 cells, and the mRNA fragment library was prepared using the NEBNext Ultra RNA Library Prep Kit for Illumina (#E7530L, New England Biolabs, Beijing, China). High‐throughput sequencing was performed by Annaroad Gene Technology (Beijing) Co., Ltd. (Beijing, China). DEGs between sh‐MEOX1 and sh‐con A2780 cells were identified based on the thresholds of |log2FC| > 1 and *p*‐value < 0.05. Finally, KEGG pathway enrichment analysis was conducted on downregulated DEGs in sh‐MEOX1 A2780 cells using a threshold of |log2FC | > 0.7, *p*‐value < 0.05.

### 4.15 Enzyme‐Linked Immunosorbent Assay (ELISA)

According to the instructions of the human S1P ELISA kit (ml038623, Mlbio, Shanghai, China), the plate was acclimatized to room temperature prior to the assay. Cell culture supernatant was collected from sh‐MEOX1 A2780, sh‐con A2780, V5‐MEOX1 SKOV3, and V5‐con SKOV3, and was added into each well with detection reagent, alongside standard samples and blank control. Then, the plate was incubated at 37°C for one hour. After thorough washing, substrates were added and incubated in the dark at 37°C for 15 min. The reaction was terminated by adding the stop solution. Finally, the OD450 value of each well was determined using a microplate reader. The concentration of S1P was calculated by interpolation from the standard curve.

### 4.16 ChIP qPCR

Following transfection with either Flag‐MEOX1 plasmids (Flag‐MEOX1 group) or the control plasmid (WT group), 293T cells were subjected to formaldehyde crosslinking. After cell lysis, the chromatin was fragmented by sonication and immunoprecipitated using anti‐Flag (#14793, Cell Signaling Technology, Danvers, MA, USA) or anti‐IgG antibodies, and then incubated with Protein A/G PLUS‐Agarose (sc‐2003, Santa Cruz, Dallas, Texas, USA). After washing successively with lysis buffer/wash buffer (100 mm Tris‐HCl, 250 mm LiCl, 1 mm EDTA, 0.5% NP40, 0.5% sodium deoxycholate, pH 8.0) and TE buffer (10 mm Tris‐HCl, 10 mm EDTA, pH 8), the beads were treated with Elution Buffer (10 mm Tris‐HCl, 10 mm EDTA, pH 8; 1% SDS) containing RNase A (ST576, Beyotime Biotechnology, Shanghai, China) and Proteinase K (ST532, Beyotime Biotechnology, Shanghai, China) to elute bound complexes and reverse crosslinks. DNA was extracted and purified for qPCR using a DNA Recovery Kit (D2110, Magen, Guangzhou, China). The promoter sequence of *SPHK1* (ENSG00000176170) was obtained from the Ensemble website (http://asia.ensembl.org/index.html). The binding sites of MEOX1 in the promoter region of *SPHK1* were predicted using the JASPAR database (https://jaspar.genereg.net/analysis). Subsequently, primers targeting these binding sites were designed with Primer Premier 5. The corresponding primer sequences are shown in Table S4.

### 4.17 Dual‐Luciferase Reporter Assay

SKOV3 cells were seeded into 24‐well plates and co‐transfected with the MEOX1 plasmid, pRL‐TK Renilla control vector, and one of the following firefly luciferase reporter constructs: pGL3‐Basic (Control), pGL3‐SPHK1 promoter (SPHK1), or pGL3‐SPHK1 mutant promoter (SPHK1‐Mut). Each transfection was carried out in triplicate wells. After 24 h, cells were harvested, and firefly and Renilla luciferase activities were measured using the Dual‐Luciferase Reporter Assay System (Beyotime Biotechnology, Shanghai, China). The relative luciferase activity was calculated as the ratio of firefly to Renilla luciferase activity, normalized to the control group.

### 4.18 Isolation of Primary NFs and CAFs

OC tissues and normal ovarian tissues were collected in DMEM medium supplemented with penicillin‐streptomycin, rinsed with pre‐cooled PBS, and minced into small sections (approximately 1 mm^3^). The tissue fragments were disseminated to the bottom of the cell culture flask at an interval of approximately 0.5 cm. Following gently adding 4 mL of complete DMEM medium, the cell flask was allowed to stand for 4 h before being inverted. Subsequently, the tissue fragments were removed after approximately one week, and the cells were cultivated until the flask was filled. The acquired cells were digested with 0.25% trypsin and then cultured in fibroblast medium. Owing to their hypersensitivity to trypsin, pure CAFs or NFs could be acquired after being passed twice according to the aforementioned method.

### 4.19 Conditioned Medium Collection

SKOV3 cells, A2780 cells, and fibroblasts were seeded in culture dishes and maintained in complete medium. After cell attachment, the medium was replaced with serum‐free medium. Following an additional 24 h of culture, the conditioned medium was collected and centrifuged at 1000 × g for 20 min. An appropriate amount of FBS was added to the supernatant to generate a complete culture medium for cell culture.

### 4.20 Collagen Gel Contraction Assays

Before preparing the gel mixture, all components were pre‐cooled to 4°C. A 500 µL gel mixture was prepared on ice, containing 5 × 10^5^ fibroblasts, 3 mg/mL rat tail collagen I (A1048301, Sigma, St. Louis, MO, USA), NaOH (1 m), PBS, and ddH_2_O according to the manufacturer's instructions. The final concentration of rat tail collagen I was adjusted to 2 mg/mL. The mixture was promptly added to a 24‐well plate and incubated at 37°C for 30 min to allow gel solidification, and then 500 µL fibroblast medium was added to each well. After gently detaching the gel from the wall and bottom of the well using a 10 µL pipette tip, the gel‐containing medium was further incubated at 37°C for 96 h. The contraction rate = gel area/well area × 100%.

### 4.21 Tube Formation Experiment

A total of 250 µL thawed Matrigel (BD356234, Becton, Dickinson and Company, New Jersey, USA) was gently added into the pre‐chilled 48‐well plate on ice, and then stored overnight at 4°C. Approximately 1.5 × 10^4^ HLECs were seeded into a 48‐well plate and cultured for 8 h at room temperature. Tube formation was observed and photographed under a Leica Microsystems CMS GmbH microscope (Leica Microsystems, Buffalo Grove, IL, USA).

### 4.22 Immunoprecipitation (IP)

HLECs were lysed in RIPA buffer containing protease and phosphatase inhibitors. Lysates (500 µg protein) were incubated with 2 µg of anti‐VEGFR3 antibody overnight at 4°C, followed by incubation with Protein A/G beads for 3 h. Beads were washed three times with RIPA buffer, and immunoprecipitated proteins were eluted by boiling in SDS sample buffer. The eluates were analyzed by immunoblotting using a pan‐phospho‐tyrosine antibody to detect phosphorylated VEGFR3, and a total VEGFR3 antibody served as the IP control. Antibodies are listed in Table S3.

### 4.23 Bioinformatics Analysis

Prognostic analysis of MEOX1, SPP1, PCDHB2, SPHK1, and S1PR3 in ovarian cancer was analyzed using the Kaplan–Meier Plotter online tool (http://kmplot.com/analysis/). Prognostic analysis of CAF infiltration in ovarian cancer was performed through the Tumor Immune Estimation Resource, version 2 (TIMER2.0, http://timer.cistrome.org/). *MEOX1* mRNA levels in different ovarian cancer cell lines were obtained from the Cancer Cell Line Encyclopedia (CCLE, https://sites.broadinstitute.org/ccle), and the mRNA levels of S1PRs in ovarian cancer were obtained from the Gene Expression Profiling Interactive Analysis version 2 online website (GEPIA2.0, http://gepia2.cancer‐pku.cn/#index). Correlation analyses between *MEOX1/SPHK1* mRNA levels and fibroblast activation markers (*FAP*, *ACTA2*, *PDGFRB*, and *S100A4*) were performed using GEPIA2.0. Correlation between *MEOX1*/*SPHK1* mRNA levels and CAF infiltration in ovarian cancer was obtained using TIMER2.0. The spatial transcriptomics dataset GSE211956 (GSM6506117_SP1/3/7/8), derived from HGSOC tissues profiled on the 10× Genomics Visium platform, was obtained from the GEO database. Cell types were annotated following previously established methods [75]. The phenoptr R package (v0.3.5) was applied to compute density–distance metrics, quantifying the spatial probability of cell occurrence relative to LECs. scRNA‐seq data from 17 ovarian cancer samples (GSE151214, GSE154600) were analyzed using the Seurat package (v4.0). Cells with 200–2500 detected genes and <20% mitochondrial reads were retained. Data were normalized and subjected to UMAP dimensionality reduction. Cell types were annotated based on canonical marker genes. *VEGFC* expression levels across cell types were compared using the Wilcoxon rank‐sum test with Bonferroni correction.

### 4.24 Statistical Analysis

All data are presented as mean ± standard deviation (S.D.). Statistical analyses were performed using the GraphPad Prism software (version 9.0). Differences between two groups were evaluated using an unpaired Student's *t‐*test, while comparisons among multiple groups were analyzed using one‐way analysis of variance (ANOVA). Categorical variables were compared using the Chi‐square test or Fisher's exact test, as appropriate. Survival analysis was conducted using the Kaplan–Meier method, and group comparisons were assessed with the log‐rank test. The correlation between the MEOX1 and SPHK1 IHC scores was examined using Pearson's correlation analysis. A *p*‐value less than 0.05 was considered statistically significant.

## Author Contributions

M.C., LQ.Y., and XL.Z. supervised the study and revised the manuscript. XT.L. contributed substantially to manuscript writing, critical revision for important intellectual content, and response to reviewers. M.C. and LQ.Y. obtained the funds. JJ.L. performed the experiments, analyzed the data, and drafted the manuscript. M.C., LQ.Y., XT.L., and XL.Z. supervised the study and revised the manuscript. XL.Z. and M.C. contributed to the data analysis. LQ.Y., M.C., YT.S., QH.L., Z.A., MY.X., Z.C., YH.S., and C.Z. contributed to the collection of clinical specimens and the corresponding clinical information.

## Funding

This study was supported by grants from the National Natural Science Foundation of China (82473110 to Liangqing Yao; 82002750 to Mo Chen), and the Shanghai Pujiang Program (23PJD009 to Mo Chen). The funding source provided financial support for this study and did not have any other involvement in this study.

## Ethics Statement

The animal experiments were approved by the Ethics Committee of Experimental Research at the Fudan University Shanghai Medical College Institutional Review Board (protocol code 202209013S, September nineth, 2022). The collection of tissue samples used in this study was approved by the Institute's Ethics Committee of the Obstetrics & Gynecology Hospital of Fudan University (protocol code 2021–94, May sixth, 2021). All written informed consents were obtained from the patients before the operation.

## Conflicts of Interest

The authors declare no conflicts of interest.

## Supporting information




**Supporting File**: advs75598‐sup‐0001‐SuppMat.docx.

## Data Availability

The data that support the findings of this study are available on request from the corresponding author. The data are not publicly available due to privacy or ethical restrictions.
