# Supplementary material for: MEOX1 Coordinates Autocrine‐Paracrine Programs via SPHK1/S1P to Promote Lymph Node Metastasis in Ovarian Cancer
Source: Adv Sci (Weinh). 2026 May 10;13(43):e23574. doi: 10.1002/advs.202523574 (PMC13336135; doi:10.1002/advs.202523574)
Supplement: Supplementary file 1 — Supporting File: advs75598‐sup‐0001‐SuppMat.docx. [file ADVS-13-e23574-s001.docx]

**Supplementary materials**

**Supplementary Figure S1-10**

**
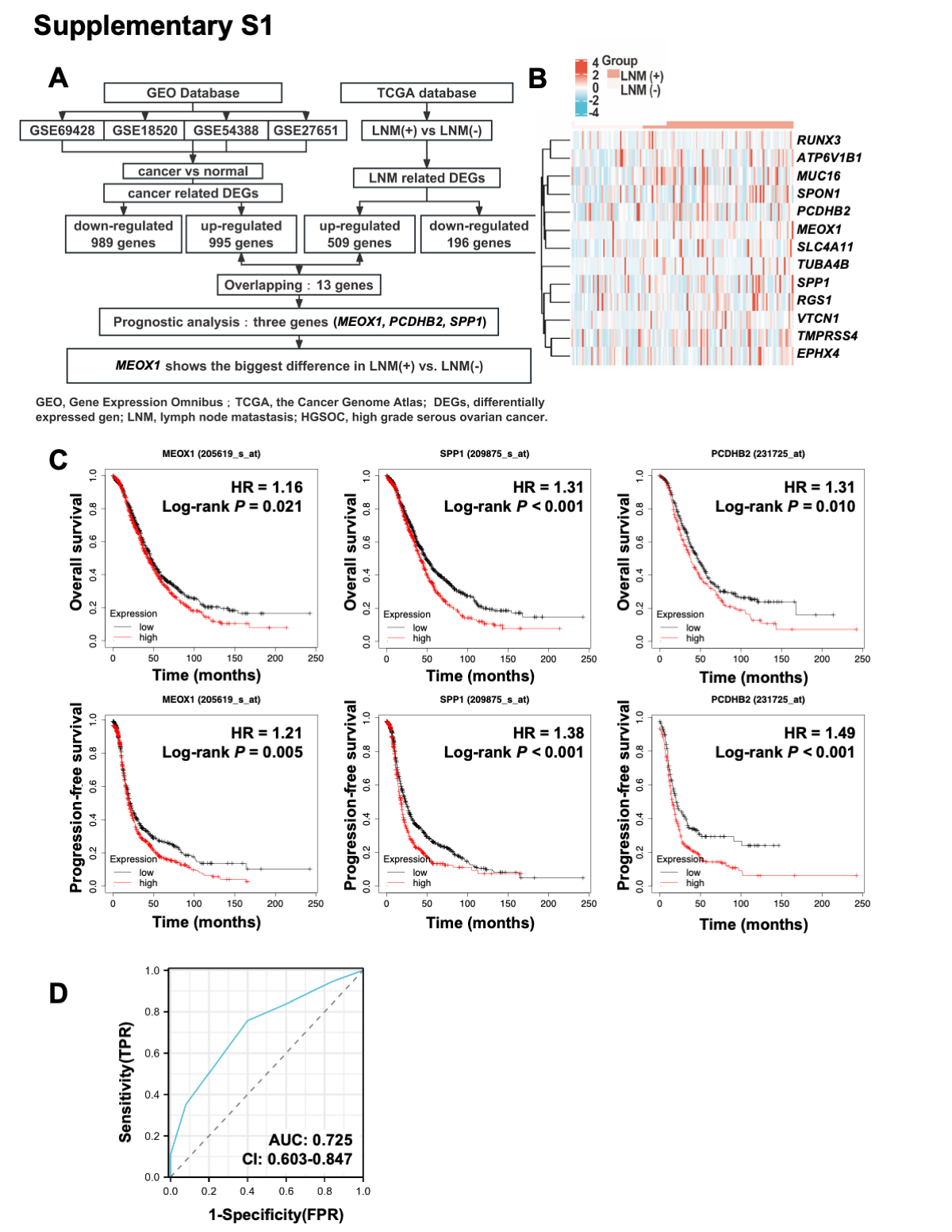
**

**Figure S1. Identification of *MEOX1* as the LNM-related gene in ovarian cancer.** A. Flow chart of the screening process for LNM-associated genes. Differentially expressed genes (DEGs) related to both cancer and LNM were identified from the GEO and TCGA databases, resulting in 13 overlapping candidates. *MEOX1* demonstrated the most significant differential expression. B. Heat map of DEGs with large differential multiples. C. Kaplan-Meier curves illustrate overall survival (OS) and progression-free survival (PFS) of patients with OC stratified by high (red) and low (black) expression of MEOX1, SPP1, and PCDHB2. Hazard ratios (HR) and log-rank *P* values are indicated for each comparison. Analysis was conducted using the Kaplan-Meier Plotter online platform (www.kmplot.com). D. ROC curve of MEOX1 IHC score in predicting LNM status of OC. AUC: the area under the curve; CI: confidence interval.The optimal cutoff value was determined to be 3.5 by maximizing Youden’s index, yielding a sensitivity of 76% and a specificity of 60%.


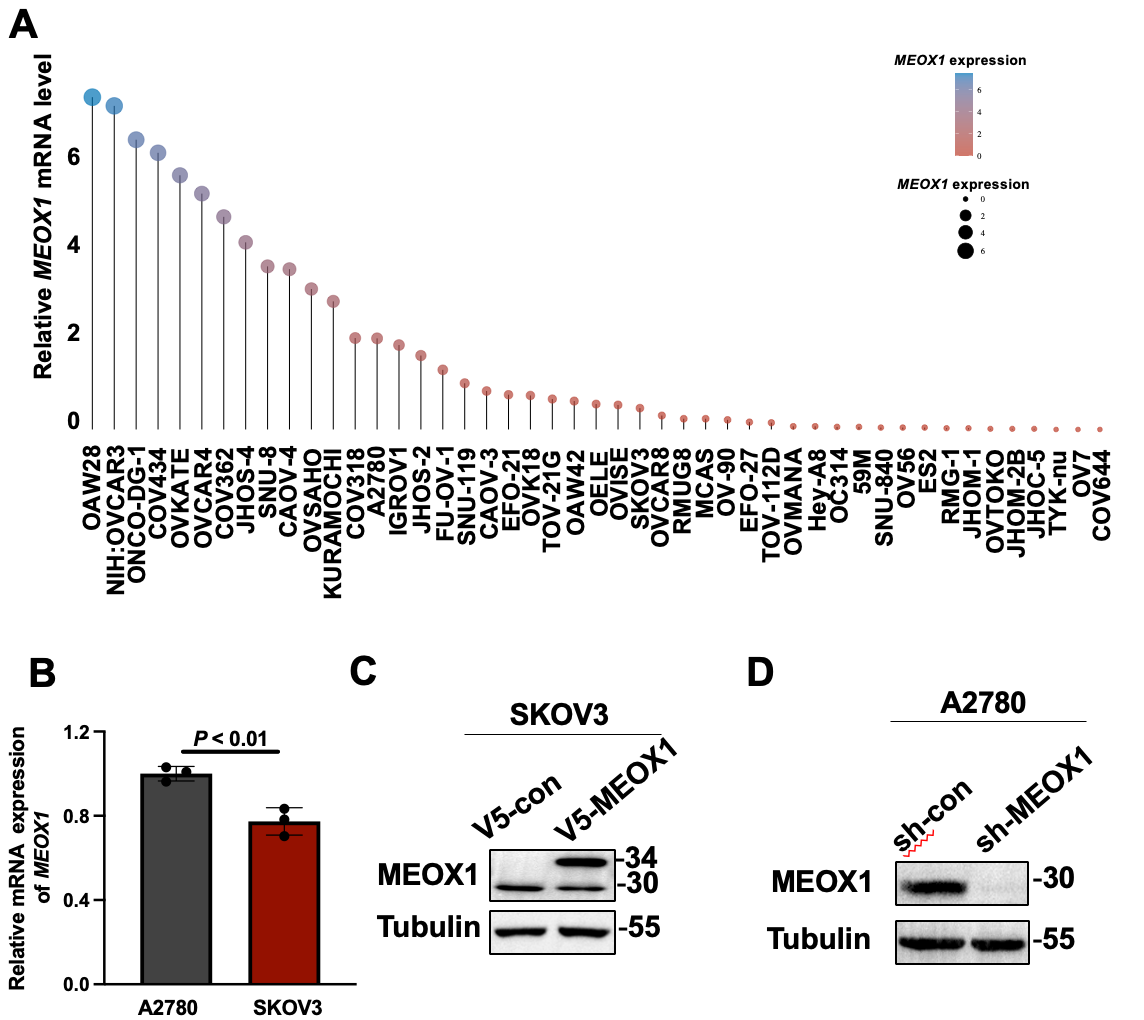


**Figure S2. Establishment of MEOX1-overexpressing or MEOX1-suppressing stable OC cell lines.** A. The relative mRNA level of *MEOX1* in various ovarian cancer cell lines derived from the CCLE database. B. RT-qPCR assays were performed to test the mRNA level of *MEOX1* in SKOV3 and A2780 cells. C-D. Western blotting assays were conducted to determine the protein expression of MEOX1 in V5-MEOX1 SKOV3 cells and V5-con SKOV3 cells (C), or in sh-MEOX1 A2780 cells and sh-con A2780 cells (D). Data are presented as mean ± SD from three independent experiments.


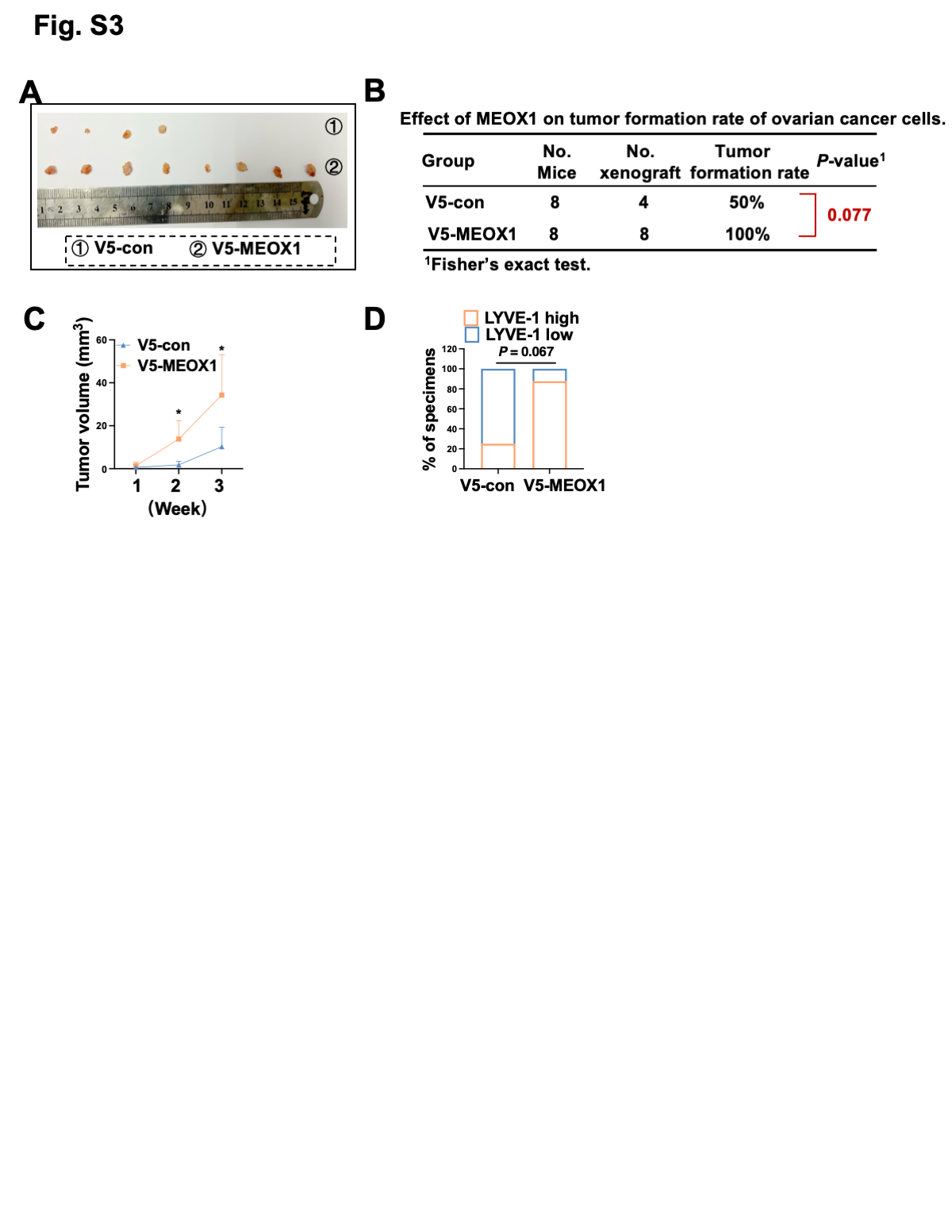


**Figure S3. MEOX1 overexpression in popliteal LNM nude mice model for ovarian cancer.** A. Images of the footpad xenografts of the V5-con and V5-MEOX1 groups in the popliteal LNM nude mice model for ovarian cancer. B. Statistical analysis of tumor formation rates in the V5-con and V5-MEOX1 groups. C. Tumor growth rates of footpad xenografts in the V5-con and V5-MEOX1 groups. Data are presented as mean ± SD. **P* < 0.05. D. The statistical data of LYVE-1 staining in V5-MEOX1 and V5-con SKOV3 groups (Chi-squared test). LYVE-1-high: >8 tubular structures per field; LYVE-1-low: ≤8 tubular structures per field (100×), averaged over 3-5 hotspot fields per case; blinded double-reading.


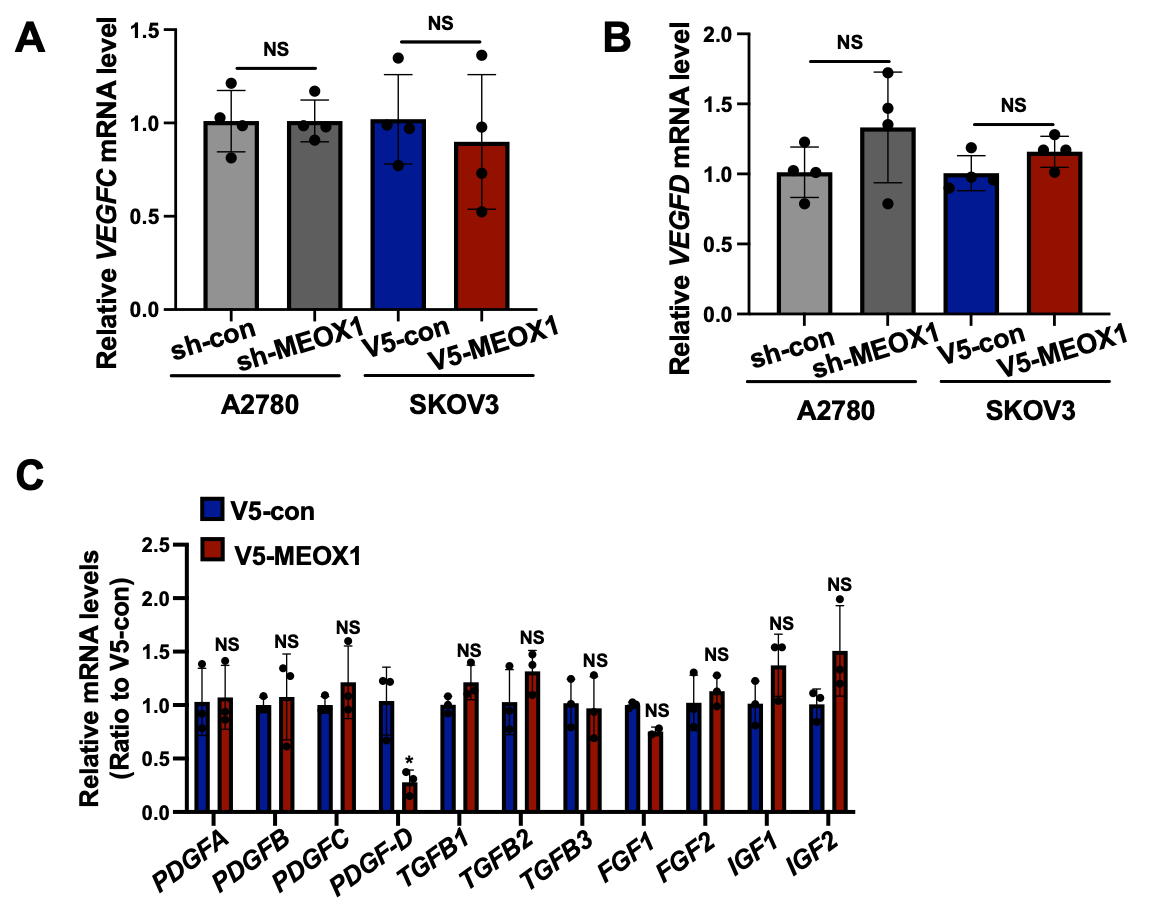


**Figure S4. MEOX1 of ovarian cancer cells did not affect lymphangiogenesis *in vitro*.** A-B. RT-qPCR assays were used to detect the mRNA changes of *VEGFC* (A) or *VEGFD* (B) after knocking down or overexpressing MEOX1 in A2780 cells or SKOV3 cells, respectively. C. RT-qPCR assays were performed to determine the mRNA alterations of *PDGFA*, *PDGFB*, *PDGFC*, and other prolymphangiogenic factors in SKOV3 cells after overexpressing MEOX1. Data are presented as mean ± SD from three independent experiments. NS, no statistical difference.


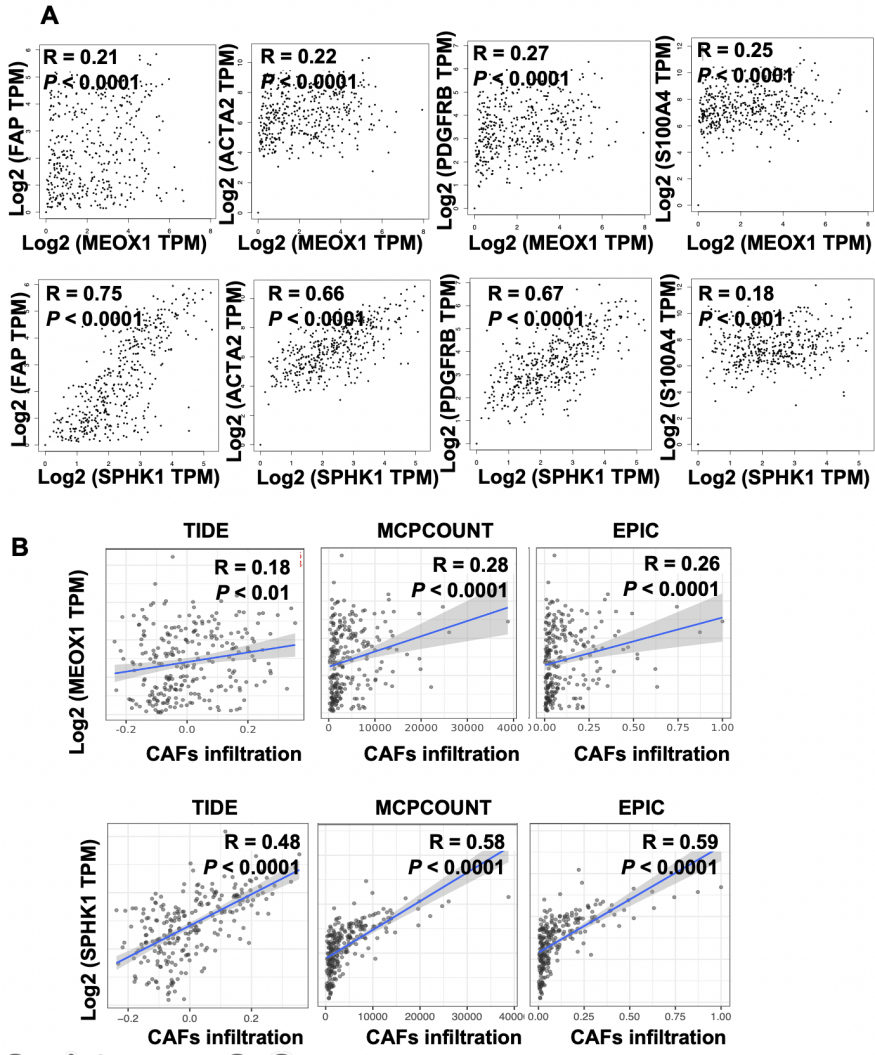


**Figure S5. Correlation analysis of MEOX1 or SPHK1 expression with the activation and infiltration of CAFs in TCGA ovarian cancer.** A. The Spearman correlation analysis of mRNA levels between *MEOX1* or *SPHK1* and CAF markers (*FAP*, *ACTA2*, *PDGFRB*, and *S100A4*) in ovarian cancer tissues, performed using the GEPIA2.0 database. B. Association of *MEOX1* or *SPHK1* mRNA expression with CAFs infiltration levels in ovarian cancer tissues, as analyzed by the TIMER2.0 database (Spearman analysis).


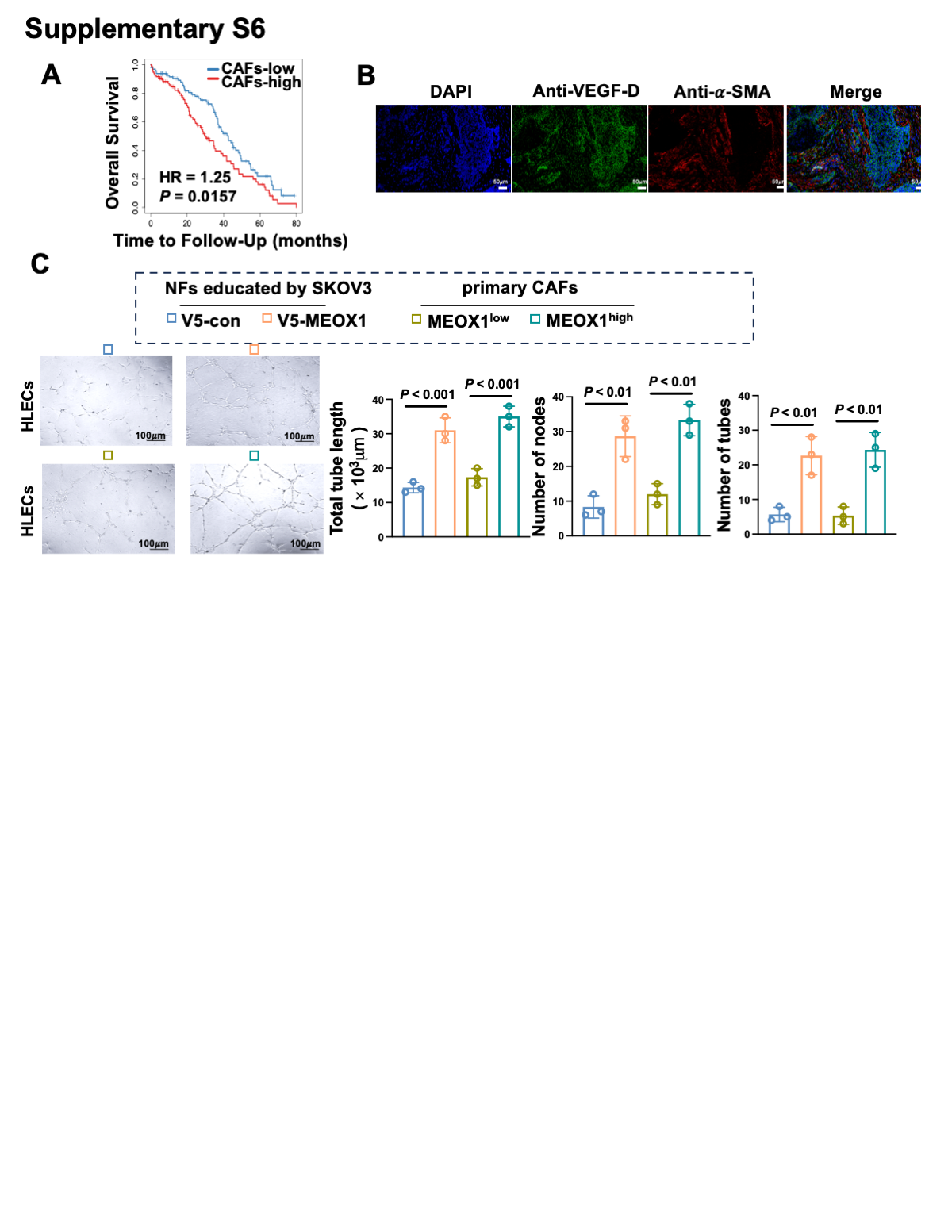


**Figure S6. MEOX1-activated CAFs promote lymphangiogenesis and correlate with poor prognosis in ovarian cancer.** A. Overall survival (OS) analysis of TCGA OC patients stratified by CAF infiltration levels, as estimated by the TIMER 2.0 database. HR, hazard ratio. B. Representative immunofluorescence co-staining images of VEGF-D and α-SMA in human OC tissue samples. C. Tube formation of HLECs treated with FCMs from NFs educated by V5-con or V5-MEOX1 SKOV3 cells or primary CAFs isolated from ovarian cancer patients with high MEOX1 expression (MEOX1^high^) or low MEOX1 expression (MEOX1^low^) for 24 hours. Right columns show the quantification of total tube length, the number of nodes, and the number of tubes. Bar graphs are presented as mean ± SD from three independent experiments.


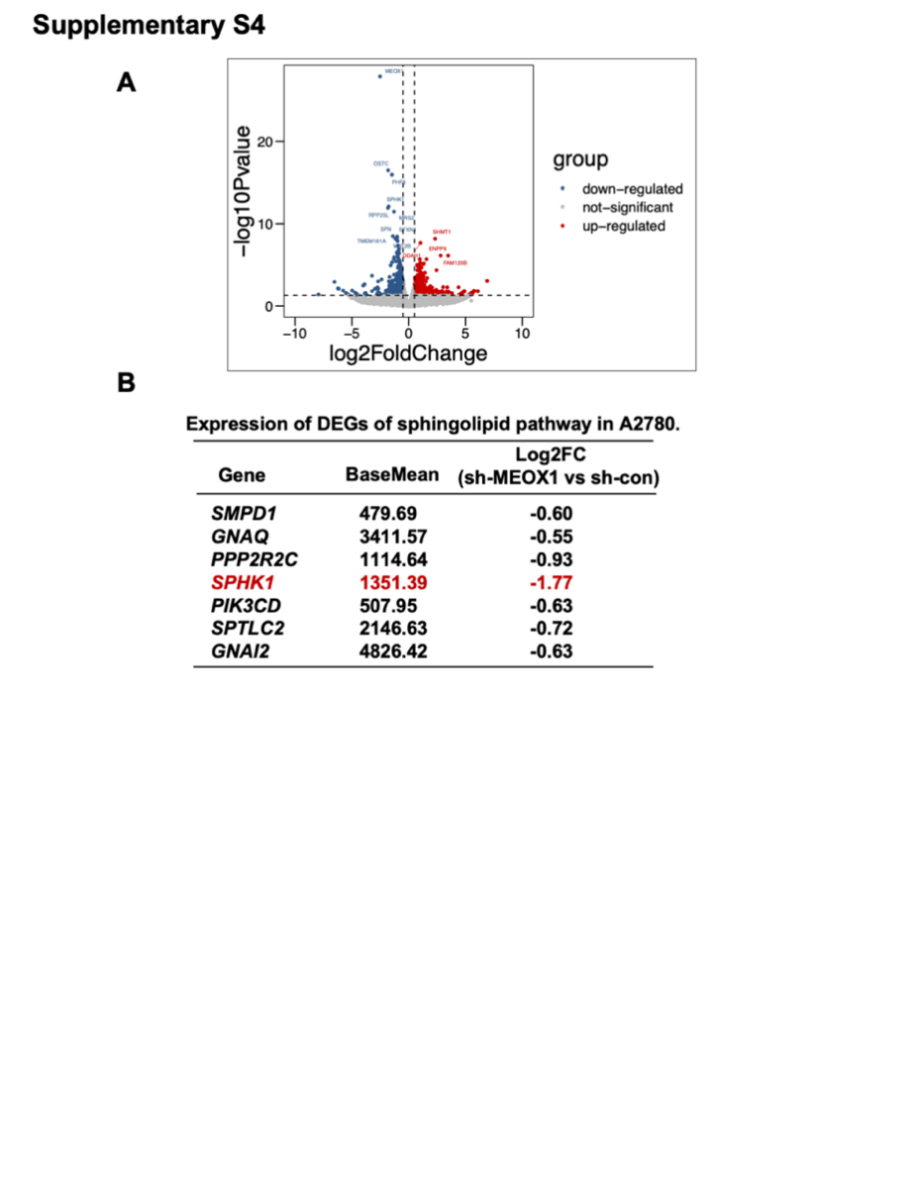


**Figure S7. Differential expression genes upon *MEOX1* knockdown in A2780 cells.** A. The volcano plot of DEGs between sh-MEOX1 and sh-con A2780 cells. B. Differential expression of genes in the sphingolipid pathway upon *MEOX1* knockdown in A2780 cells.


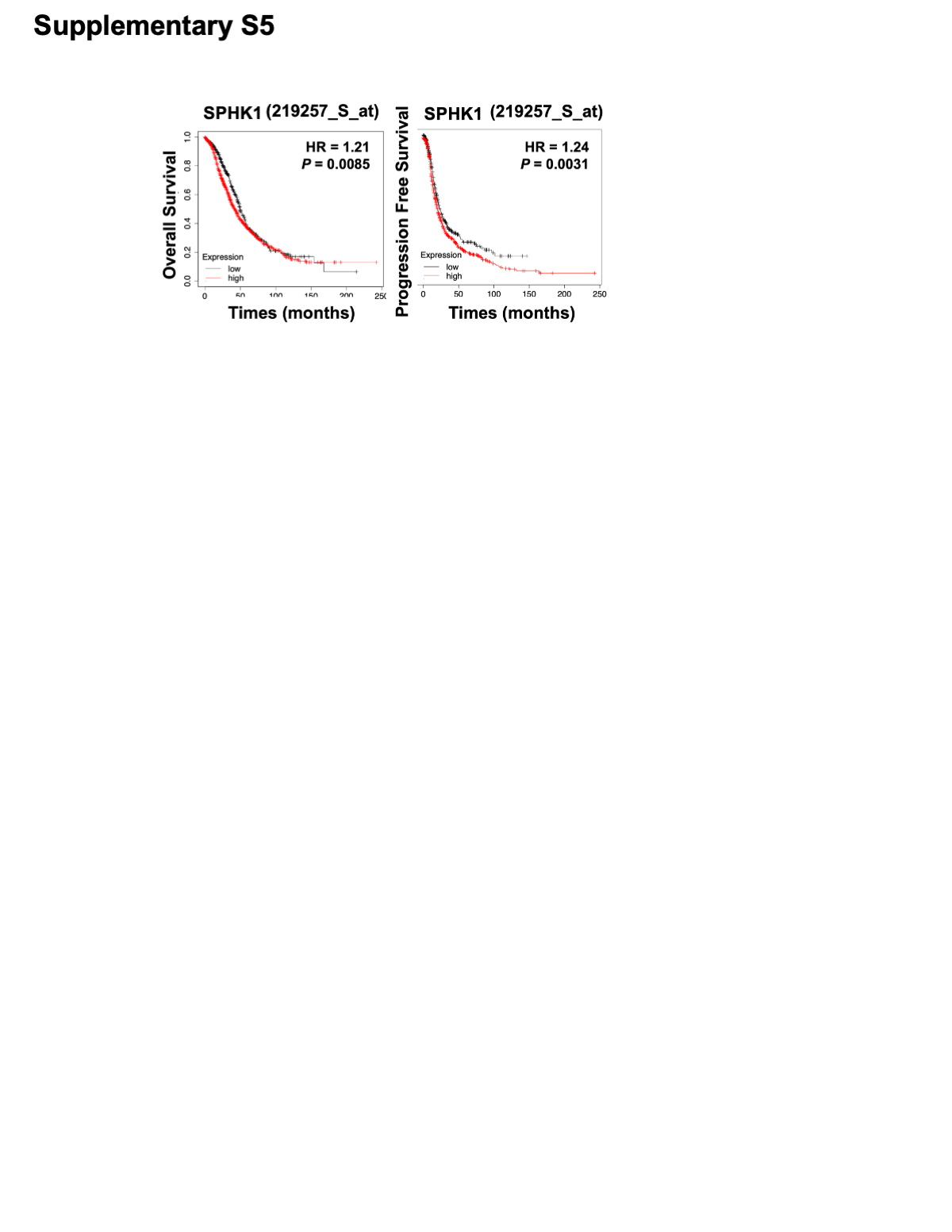


**Figure S8. Kaplan Meier Plotter analysis of SPHK1 expression with OS (*Left*) and PFS (*Right*) in OC.**


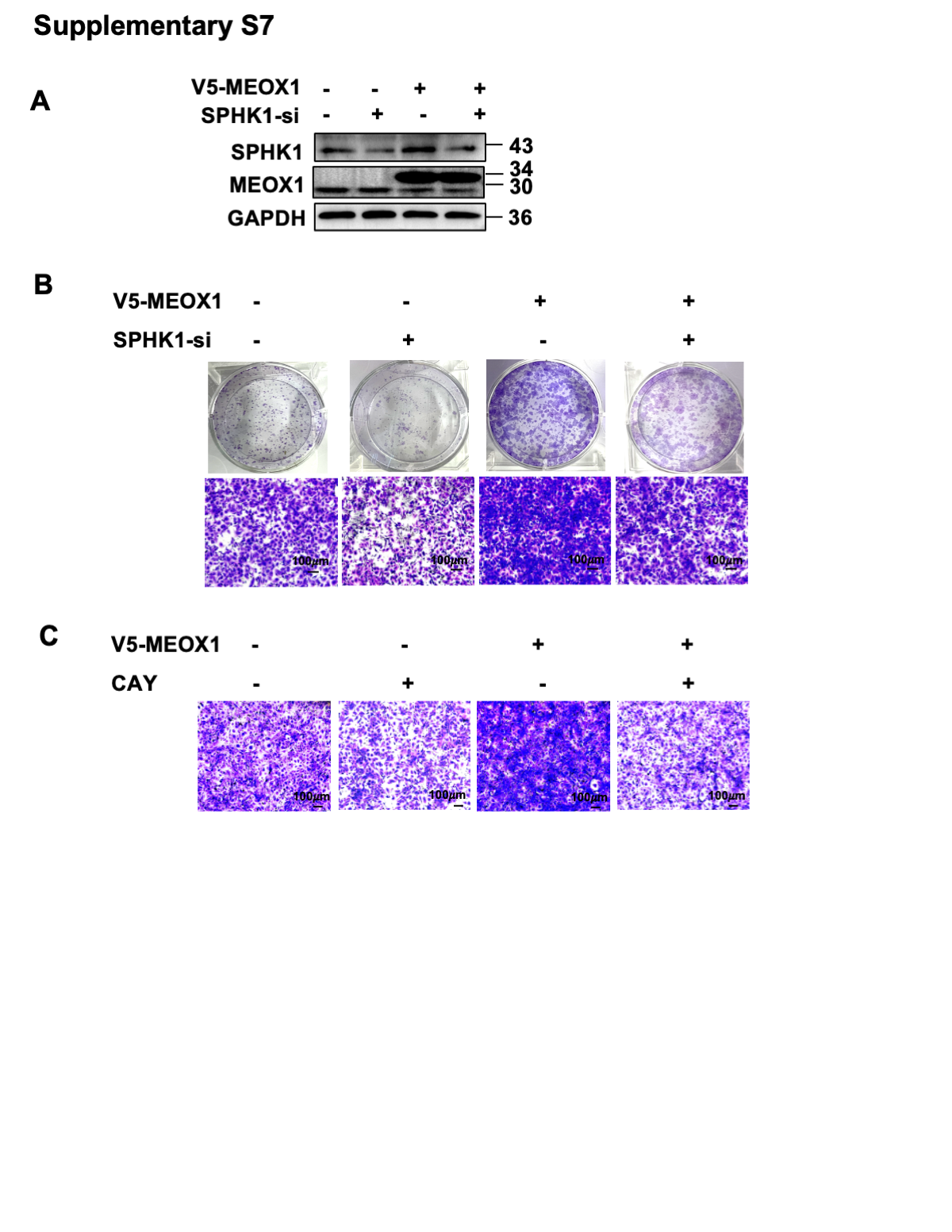


**Figure S9. SPHK1 knockdown or the S1PR3 inhibitor reversed the promotion of ovarian cancer cell proliferation and migration induced by MEOX1 overexpression.** A. SPHK1-siRNA or NC-siRNA were transfected into V5-MEOX1 SKOV3 and V5-con SKOV3 cells for 24 hours and then the protein expression of MEOX1 and SPHK1 was detected. B. Representative images of colony proliferation assays (*upper*) and Transwell migration assays (*lower)*. Both assays were performed using V5-con and V5-MEOX1 SKOV3 cells transfected with NC-siRNA or SPHK1-siRNA. C. Representative staining images showing migrated cells of V5-con or V5-MEOX1 SKOV3 cells treated with or without 20 μM CAY10444 for 24 hours.


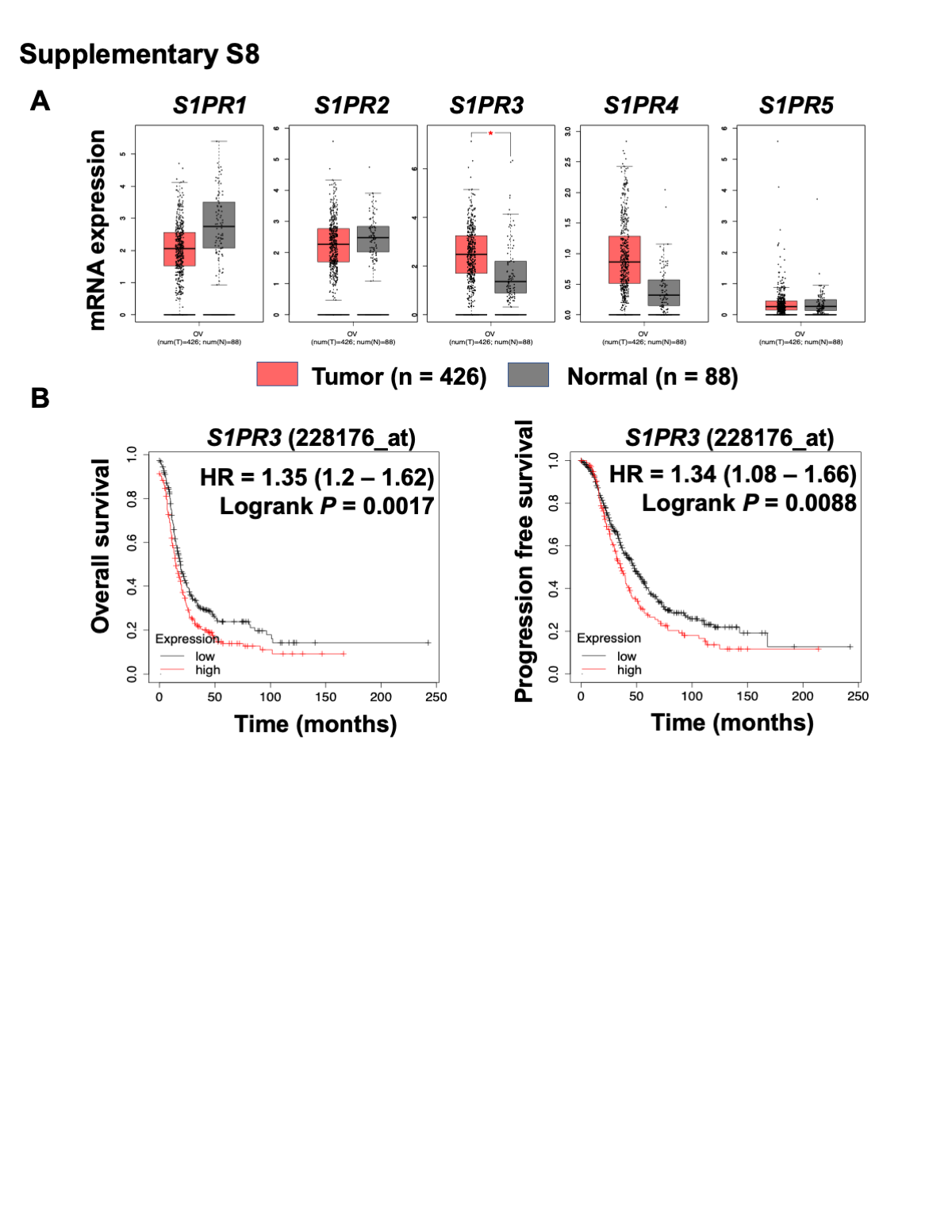


**Figure S10. The mRNA expression levels of S1PRs and prognostic analysis of S1PR3 in ovarian cancer.** A. The mRNA levels of *S1PR1*, *S1PR2*, *S1PR3*, *S1PR4*, and *S1PR5* in ovarian cancer tissues and normal ovarian tissues, as analyzed using the GEPIA 2.0 database. B. Association between S1PR3 expression and OS or PFS of ovarian cancer patients, evaluated via the Kaplan Meier Plotter online tool.


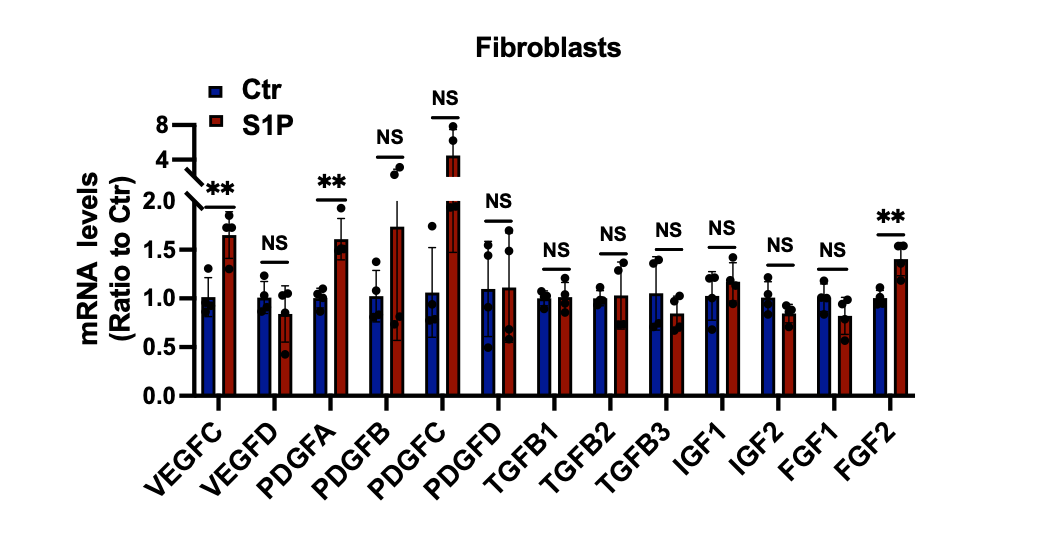


**Figure S11. The effect of S1P on the expression of prolymphangiogenic factors in fibroblasts.** RT-qPCR assays were performed to test the mRNA expression of prolymphangiogenic factors in NFs treated with or without 0.1 μM S1P for 24 hours. Data are presented as mean ± SD from four independent experiments. NS, no statistical difference; ***P* < 0.01.


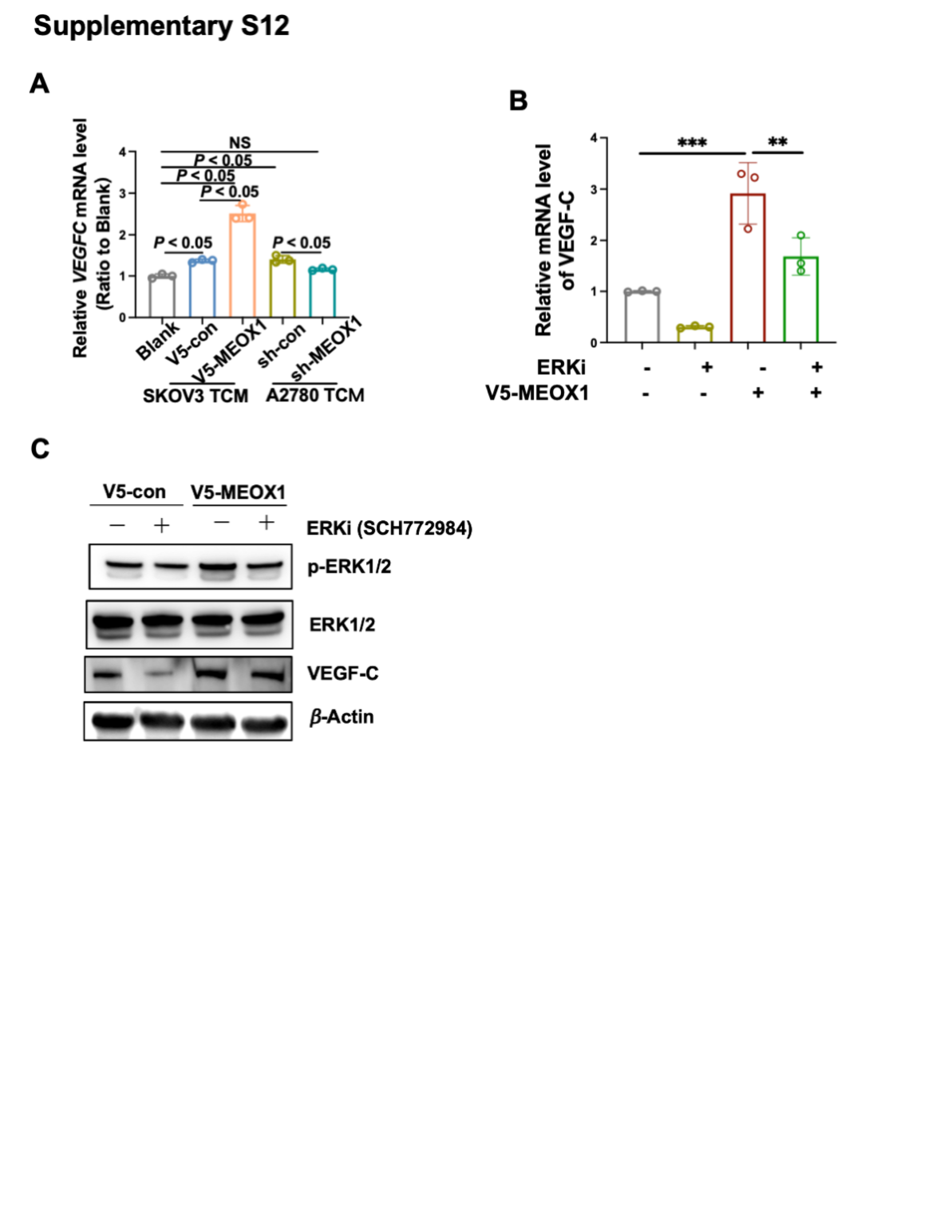


**Figure S12. MEOX1 promotes fibroblast VEGF-C expression via ERK activation.** A. RT-qPCR assays were performed to detect mRNA levels of VEGFC in NFs after 5-day treatment with TCM from SKOV3 cells (V5-con vs. V5-MEOX1) or A2780 cells (sh-con vs. sh-MEOX1). B. RT-qPCR analysis of *VEGFC* mRNA levels in NFs cultured with TCM from V5-con or V5-MEOX1 SKOV3 in the presence or absence of the ERK inhibitor SCH772984 (100 nM) for 24 hours. Data are presented as mean ± SD from three independent experiments. ***P* < 0.01, ****P* < 0.001. C. Western blot analysis of VEGF-C, p-ERK1/2, and total ERK1/2 in NFs treated as in (B).


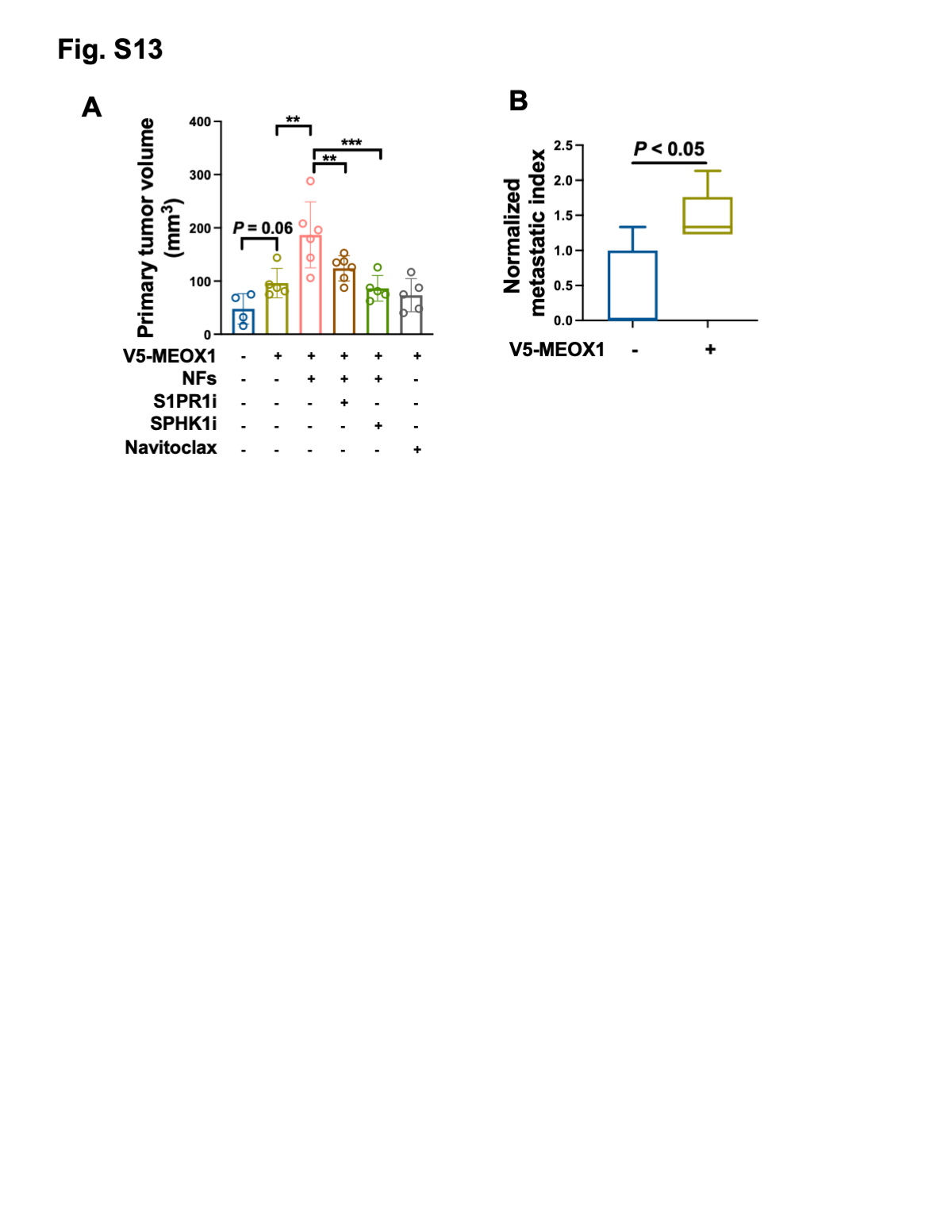


**Figure S13. MEOX1 promotes lymph node metastasis beyond its effect on primary tumor volume *in vivo*. A. Primary tumor volumes from footpad xenografts of the indicated groups: V5-con+Vehicle, V5-MEOX1+Vehicle, V5-MEOX1+NFs+Vehicle, V5-MEOX1+NFs+S1PR1i, V5-MEOX1+NFs+SPHK1i, and V5-MEOX1+Navitoclax. Data are presented as mean ± SD. ***P* < 0.01, ****P* < 0.001. B. Normalized metastatic index (number of metastatic lymph nodes/ primary tumor volume (mm³)) in V5-con+Vehicle and V5-MEOX1+Vehicle groups.**

**Supplementary Table S1-4**

**Table S1. The average expression and fold-change of *MEOX1*, *PCDHB2*, and *SPP1* between the LNM (+) and LNM (-) TCGA ovarian cancer**

| **Gene** | **Log2FC** | **AveExpr** | ***P-*Value** |
| --- | --- | --- | --- |
| *MEOX1* | 0.823765948 | 2.852735426 | 0.034216191 |
| *PCDHB2* | 0.726192974 | 2.599744727 | 0.045329482 |
| *SPP1* | 0.682018949 | 8.396926678 | 0.008022262 |

**Table S2. The clinical and pathological characteristics of advanced ovarian cancer patients with or without lymph node metastasis**

| **Variable** | **LNM(-)**  **(n = 25)** | **LNM(+)**  **(n = 37)** | ***P*^1^** |
| --- | --- | --- | --- |
|  | **n (%)** | **n (%)** |  |
| **Age (years)** |  |  | 0.789 |
| < 50 | 6 (24.0) | 10 (27.0) |  |
| ≥ 50 | 19 (76.0) | 27 (73.0) |  |
| **FIGO Stage** |  |  | 0.719 |
| III | 11 (44.0) | 18 (48.6) |  |
| IV | 14 (56.0) | 19 (51.4) |  |
| **Histological subtype** |  |  | 0.335 |
| Serous Adenocarcinoma | 21 (84.0) | 34 (97.3) |  |
| Others | 4 (16.0) | 3 (2.7) |  |
| **Tumor size (cm^3^)** |  |  | 0.171 |
| < 1000 | 20 (76.0) | 34 (97.3) |  |
| ≥ 1000 | 5 (24.0) | 3 (2.7) |  |
| **CA125 level (U/ml)** |  |  | 0.712 |
| < 600 | 12 (48.0%) | 16 (43.2) |  |
| ≥ 600 | 13 (42.0%) | 21 (56.8) |  |
| **Ascites** |  |  |  |
| Absent | 6 (24.0) | 13 (35.1) | 0.351 |
| Present | 19 (76.7) | 24 (64.9) |  |

^1^Chi-square test

**Table S3. Antibodies used for IHC, immunofluorescence staining, and western blot**

| **Antibody** | **Item No.** | **Manufacturer** |
| --- | --- | --- |
| anti-MEOX1(for IHC) | ab279366 | Abcam, Cambridge, UK |
| anti-LYVE-1 | NB600-1008 | Novus Biologicals, Littleton, Colorado, USA |
| anti-SPHK1 | ab262697 | Abcam, Cambridge, UK |
| anti-α-SMA(Rabbit) | ab124964 | Abcam, Cambridge, UK |
| anti-FAP | ab207178 | Abcam, Cambridge, UK |
| anti-CK7 | ab199718 | Abcam, Cambridge, UK |
| anti-α-SMA (Mouse) | BM0002 | Boster, Wuhan, China |
| anti-VEGF-C | 22601-1-AP | Proteintech, Rosemont, IL, USA |
| anti-VEGF-D | 26915-1-AP | Proteintech, Rosemont, IL, USA |
| anti-MEOX1 (for WB) | TA804716 | Origene, Wuxi, China |
| anti-SPHK1 | ab262697 | Abcam, Cambridge, UK |
| anti-MMP2 | ab92536 | Abcam, Cambridge, UK |
| anti-MMP9 | ab76003 | Abcam, Cambridge, UK |
| anti-Vimentin | #5741 | Cell Signaling Technology, Danvers, MA, USA |
| anti-ERK1/2 | #9102 | Cell Signaling Technology, Danvers, MA, USA |
| anti-P-ERK1/2 | #4370 | Cell Signaling Technology, Danvers, MA, USA |
| anti-Tubulin | 11224-1-AP | Proteintech, Rosemont, IL, USA |
| anti-GAPDH | #5174 | Cell Signaling Technology, Danvers, MA, USA |
| secondary antibody goat anti-rabbit | 31460 | Invitrogen, Carlsbad, CA, USA |
| secondary antibody goat anti-mouse | 31430 | Invitrogen, Carlsbad, CA, USA |
| Alexa Fluor 488 labelled donkey anti-rabbit IgG | ab150073 | Abcam, Cambridge, UK |
| Alexa Fluor 594 labelled donkey anti-rabbit IgG | 711-585-152 | Jackson, West Grove, PA, USA |
| Alexa Fluor 488 labelled donkey anti-mouse IgG | 715-545-151 | Jackson, West Grove, PA, USA |
| Alexa Fluor 594 labelled donkey anti-mouse IgG | ab150108 | Abcam, Cambridge, UK |

**Table S3. (Continued)**

| **Antibody** | **Item No.** | **Manufacturer** |
| --- | --- | --- |
| CY3-conjugated goat anti-rabbit IgG | GB21303 | Servicebio, Wuhan, China |
| S-vision IHC polymer secondary antibody (goat anti-rabbit) | G1302 | Servicebio, Wuhan, China |
| Anti-CK7 | GB15225 | Servicebio, Wuhan, China |
| Anti-VEGFR3 | HA724191 | Huabio, Hangzhou, China |
| Anti-Phospho-(Ser/Thr) Phe | #9631 | Cell Signaling Technology, Danvers, MA, USA |

**Table S4. Gene primers used for RT-qPCR and ChIP qPCR**

| **Primer** | **Sequence (5’-3’)** |
| --- | --- |
| *MEOX1*-F | GCAGGGGGTTCCAAGGAAA |
| *MEOX1*-R | GTCAGGTAGTTATGATGGGCAAA |
| *SPHK1*-F | GCTCTGGTGGTCATGTCTGG |
| *SPHK1*-R | CACAGCAATAGCGTGCAGT |
| *S1PR1*-F | TTCCACCGACCCATGTACTAT |
| *S1PR1*-R | GCGAGGAGACTGAACACGG |
| *S1PR2*-F | CATCGTCATCCTCTGTTGCG |
| *S1PR2*-R | GCCTGCCAGTAGATCGGAG |
| *S1PR3*-F | CGGCATCGCTTACAAGGTCAA |
| *S1PR3*-R | GCCACGAACATACTGCCCT |
| *VEGFC*-F | GGCTGGCAACATAACAGAGAA |
| *VEGFC*-R | CCCCACATCTATACACACCTCC |
| *VEGFD*-F | TCCCATCGGTCCACTAGGTTT |
| *VEGFD*-R | AGGGCTGCACTGAGTTCTTTG |
| *PDGFA*-F | GCAAGACCAGGACGGTCATTT |
| *PDGFA*-R | GGCACTTGACACTGCTCGT |
| *PDGFB*-F | CTCGATCCGCTCCTTTGATGA |
| *PDGFB*-R | CGTTGGTGCGGTCTATGAG |
| *PDGFC*-F | ATTCACAGCCCAAGGTTTCCT |

**Table S4. (Continued)**

| **Primer** | **Sequence (5’-3’)** |
| --- | --- |
| *PDGFC*-R | GGGTCTTCAAGCCCAAATCTT |
| *PDGFD*-F | TTGTACCGAAGAGATGAGACCA |
| *PDGFD*-R | GCTGTATCCGTGTATTCTCCTGA |
| *TGFB1*-F | GGCCAGATCCTGTCCAAGC |
| *TGFB1*-R | GTGGGTTTCCACCATTAGCAC |
| *TGFB2*-F | CAGCACACTCGATATGGACCA |
| *TGFB2*-R | CCTCGGGCTCAGGATAGTCT |
| *TGFB3*-F | ACTTGCACCACCTTGGACTTC |
| *TGFB3*-R | GGTCATCACCGTTGGCTCA |
| *FGF1*-F | CTCCCGAAGGATTAAACGACG |
| *FGF1*-R | GTCAGTGCTGCCTGAATGCT |
| *FGF2*-F | AGAAGAGCGACCCTCACATCA |
| *FGF2*-R | CGGTTAGCACACACTCCTTTG |
| *IGF1*-F | GCTCTTCAGTTCGTGTGTGGA |
| *IGF1*-R | GCCTCCTTAGATCACAGCTCC |
| *IGF2*-F | GTGGCATCGTTGAGGAGTG |
| *IGF2*-R | CACGTCCCTCTCGGACTTG |
| *GAPDH*-F | GGAGCGAGATCCCTCCAAAAT |
| *GAPDH*-R | GGCTGTTGTCATACTTCTCATGG |
| Binding site1-ChIP-F | CGAACTCACAGTGCTCCCTGTG |
| Binding site1-ChIP-R | GGTACTGGCTGCGGAGATTAGC |
| Binding site2-ChIP-F | GGTCCGTCCGGAAGAGAAGACAC |
| Binding site2-ChIP-R | CCCACACACCTCTCTCATCGC |
